# Locomotor- and Reward-Enhancing Effects of Cocaine Are Differentially Regulated by Chemogenetic Stimulation of Gi-Signaling in Dopaminergic Neurons

**DOI:** 10.1523/ENEURO.0345-17.2018

**Published:** 2018-06-18

**Authors:** Annika H. Runegaard, Andreas T. Sørensen, Ciarán M. Fitzpatrick, Søren H. Jørgensen, Anders V. Petersen, Nikolaj W. Hansen, Pia Weikop, Jesper T. Andreasen, Jens D. Mikkelsen, Jean-Francois Perrier, David Woldbye, Mattias Rickhag, Gitta Wortwein, Ulrik Gether

**Affiliations:** 1 Molecular Neuropharmacology and Genetics Laboratory, Department of Neuroscience, Faculty of Health and Medical Sciences, University of Copenhagen, Copenhagen, DK-2200, Denmark; 2 Department of Drug Design and Pharmacology, Faculty of Health and Medical Sciences, University of Copenhagen, Copenhagen, DK-2100, Denmark; 3 Neuronal Signaling Laboratory, Department of Neuroscience, Faculty of Health and Medical Sciences, University of Copenhagen, Copenhagen, DK-2200, Denmark; 4 Laboratory of Neuropsychiatry, Psychiatric Center Copenhagen, University of Copenhagen, Copenhagen, DK-2100, Denmark; 5 Neurobiology Research Unit, Copenhagen University Hospital, Rigshospitalet, Copenhagen, DK-2100, Denmark

**Keywords:** addiction, chemogenetics, cocaine, dopamine, G protein–coupled receptors, mouse behavior

## Abstract

Dopamine plays a key role in the cellular and behavioral responses to drugs of abuse, but the implication of metabotropic regulatory input to dopaminergic neurons on acute drug effects and subsequent drug-related behavior remains unclear. Here, we used chemogenetics [Designer Receptors Exclusively Activated by Designer Drugs (DREADDs)] to modulate dopamine signaling and activity before cocaine administration in mice. We show that chemogenetic inhibition of dopaminergic ventral tegmental area (VTA) neurons differentially affects locomotor and reward-related behavioral responses to cocaine. Stimulation of Gi-coupled DREADD (hM4Di) expressed in dopaminergic VTA neurons persistently reduced the locomotor response to repeated cocaine injections. An attenuated locomotor response was seen even when a dual-viral vector approach was used to restrict hM4Di expression to dopaminergic VTA neurons projecting to the nucleus accumbens. Surprisingly, despite the attenuated locomotor response, hM4Di-mediated inhibition of dopaminergic VTA neurons did not prevent cocaine sensitization, and the inhibitory effect of hM4Di-mediated inhibition was eliminated after withdrawal. In the conditioned place-preference paradigm, hM4Di-mediated inhibition did not affect cocaine-induced place preference; however, the extinction period was extended. Also, hM4Di-mediated inhibition had no effect on preference for a sugar-based reward over water but impaired motivation to work for the same reward in a touchscreen-based motivational assay. In addition, to support that VTA dopaminergic neurons operate as regulators of reward motivation toward both sugar and cocaine, our data suggest that repeated cocaine exposure leads to adaptations in the VTA that surmount the ability of Gi-signaling to suppress and regulate VTA dopaminergic neuronal activity.

## Significance Statement

The addictive properties of cocaine and other drugs of abuse are tightly coupled to altered dopamine (DA) neurotransmission mediated by dopaminergic neurons in the ventral tegmental area (VTA). Here, we used DREADDs to investigate how modulation of these neurons via G protein–coupled receptors (GPCRs) affects the behavioral effects of cocaine. The data substantiate dopaminergic VTA neurons as a prime regulator of explorative locomotion and reward motivation, as well as reveal a delicate role of metabotropic Gi-coupled input with differential effects during acute, repeated, and sensitized responses to cocaine. The results may provide an important framework for exploring new principles for addiction treatment via modulation of GPCR signaling within the dopaminergic system.

## Introduction

Addictive drugs all share the common property of causing elevation of extracellular dopamine (DA) in brain regions related to motor and limbic functions (Di Chiara and Imperato, 1988). Cocaine elicits its main stimulatory effects by blocking the reuptake of DA via the DA transporter (DAT), leading to a rapid increase in extracellular DA levels in areas receiving dopaminergic innervation (Ritz et al., 1987; Chen et al., 2006; Torres and Amara, 2007; Durieux et al., 2012). Although cocaine acts with similar affinity on both the serotonin and noradrenergic transporters, the behavioral effects of cocaine have been assigned primarily to altered DA transmission (Chen et al., 2006; Torres and Amara, 2007; Kristensen et al., 2011). Indeed, seminal work from the 1980s and 1990s, together with more recent work, have substantiated an unambiguous role of DA in cocaine’s actions, including not only the immediate locomotor-enhancing response but also cocaine-induced behavioral sensitization and reward-enhancing effects (Roberts and Koob, 1982; Giros and Caron, 1993; Chen et al., 2006; Tilley et al., 2009). In the striatum, increased synaptic levels of DA caused by cocaine lead to enhanced postsynaptic DA receptor signaling as well as stimulation of presynaptic G protein–coupled DA D_2_ autoreceptors, providing a modulatory feedback loop that enables tight control of release and firing of DA neurons (Beckstead et al., 2004; Beckstead and Williams, 2007). It is therefore not surprising that D_2_ autoreceptors have been shown to influence locomotion, reward-processing, drug-cue association, and relapse (Baik, 2013; Ford, 2014). Furthermore, VTA DA neurons receive inhibitory metabotropic inputs via Gi-coupled glutamate, GABA, and endocannabinoid G protein–coupled receptors (GPCRs; Johnson and Lovinger, 2016). Their activity, together with ionotropic inputs, adjusts the firing rate of DA neurons and DA release and transmission and has been demonstrated to influence the behavioral response to cocaine (Glangetas et al., 2015; Johnson and Lovinger, 2016; Edwards et al., 2017). These results not only support the assumption that cocaine requires dopaminergic neuronal activity to increase extracellular DA (given the re-uptake inhibition by cocaine) but also suggest strong influence of GPCR signaling in mesolimbic DAergic neurons on the behavioral responses to drugs like cocaine. Thus, GPCR signaling in DA neurons may have major impact on acute cocaine-induced responses, possibly influencing individual responses to cocaine and vulnerability to drug addiction (Johnson and Lovinger, 2016).

To better understand how modulation of VTA DA signaling and activity affects the acute effects of cocaine and cocaine reward perception and contributes to the transition toward addiction, we employed a chemogenetic approach and expressed Designer Receptors Exclusively Activated by Designer Drugs (DREADDs; Armbruster et al., 2007; Ferguson and Neumaier, 2015; Roth, 2016) in DA neurons of the VTA. Specifically, we took advantage of the Gi-coupled hM4Di, which has been shown to reduce phasic firing of neurons by inducing membrane hyperpolarization through reduced cyclic adenosine monophosphate (cAMP) signaling and activation of G protein–coupled inwardly rectifying potassium (GIRK) channels (Armbruster et al., 2007; Stachniak et al., 2014). Thus, hM4Di was used as a tool to assess how transient reduction of VTA DA activity via inhibitory GPCR signaling cascades affects locomotor and reward-related behaviors in mice, before and after acute and repeated cocaine exposures. Our data substantiate the delicate role of VTA dopaminergic neurons on explorative locomotion and reward motivation but not reward perception, as also indicated by irreversible lesion studies (Kelly et al., 1975; Roberts and Koob, 1982; Berridge et al., 1989; Berridge and Robinson, 2016). We specifically provide evidence that repeated exposure to cocaine leads to major neuronal adaptations in the VTA that overcome the ability of inhibitory metabotropic input to alter VTA DA-driven behaviors, including cocaine-induced locomotion and reward. Furthermore, we find that while inhibition of VTA dopaminergic neurons has no influence on the expression of cocaine-induced conditioned place preference, it does affect subsequent drug-seeking behavior. Likewise, inhibition of VTA dopaminergic neurons had no effect on the consumption of, and preference for, a sugar reward, but significantly impaired the motivation to work for that reward in a touchscreen-based assay. These results demonstrate that VTA dopaminergic neurons operate as critical regulators of reward motivation toward both sugar and cocaine.

## Materials and Methods

### Mice

TH-Cre mice (Savitt et al., 2005) were bred in-house. Male mice were used for behavioral experiments, while both male and female mice were used for immunohistochemistry and electrophysiological recordings. All experiments were performed in accordance with institutional guidelines and regulations.

### Drugs

Cocaine hydrochloride was dissolved in saline (0.9%). CNO (Tocris) was dissolved in DMSO (Sigma-Aldrich) and diluted in saline for a final concentration of DMSO of 0.4%. Both cocaine and CNO were diluted for a final intraperitoneal (i.p.) administration of 10 ml/kg bodyweight. Equivalent volumes of saline and CNO vehicle (VEH: 0.4% DMSO) were injected in control animals.

### Stereotaxic injections and AAV constructs

Adult wild-type (WT) and hemizygous TH-Cre mice (8–16 wk old) were bilaterally injected into the VTA (from bregma: AP –3.3 mm, ML ± 0.5 mm, from skull DV: 4.5 mm) with adeno-associated virus (AAV) vectors encoding a Cre-dependent DREADD or control transgene: AAV8-hSyn-DIO-mCherry, AAV8-hSyn-DIO-hM4Di-mCherry, or AAV8-hSyn-DIO-rM3Ds-mCherry (Roth, 2016; Vector Core at University of North Carolina). For projection-specific targeting, a dual-viral approach was applied; a Cre-dependent Flp transgene, delivered by retrograde canine adenovirus 2 (CAV2; CAV2-CMV-DIO-Flp; Schwarz et al., 2015; Biocampus), was injected into the ventral striatum (NAc; AP: 1.00, ML: 1.25, DV: –4.5), whereas a Flp-dependent DREADD transgene, delivered by AAV (AAV8-hSyn-fDIO-hM4Di-mCherry), was injected into the VTA. This AAV was manufactured in-house according to the protocol described previously (Sørensen et al., 2016).

Viral injections were conducted under general isoflurane anesthesia in a stereotaxic instrument (Kopf Instruments). Saline droplets were applied to the eyes to prevent drying. A midline incision was made down the scalp, and a craniotomy was made using a dental drill for bilateral infusions. On each side, a volume of 300 nl AAV (titers >10^12^) was manually injected over 5 min using a glass cannula coupled to a 10-μl Hamilton syringe, which was withdrawn 3 min after the end of the infusion. Before stereotaxic surgery and for 2 d after, mice were subcutaneously administered an analgesic and antibiotic mixture of Rimadyl and Baytril (0.5 mg/ml Rimadyl and 1 mg/ml Baytril in saline) at 10 ml/kg body weight. The incision was closed with absorbable suture, and the mice were given a subcutaneous saline injection to remain hydrated postsurgery, before being placed in a cage with heating pads to recover. For the behavioral studies, each cohort of mice contained an additional two to three DREADD-injected mice to account for any exclusions. At the end, 4 of 145 surgerized DREADD mice were excluded after evaluating viral expression after end experiments. Behavioral experiments were initiated after recovery ∼3 wk postinjections.

### Expression verification and histology

Three weeks postinjection, mice injected with AAvs carrying mCherry, hM4Di-mCherry, or rM3Ds-mCherry were anesthetized with pentobarbital and transcardially perfused with cold 4% paraformaldehyde (PFA) in 0.1 m PBS. Expression of mCherry was assessed by immunohistochemistry in coronal sections (40 μm) from the striatum and midbrain. Sections were incubated for 20 min in 0.01 m PBS with 0.3% Triton X-100, 5% swine serum, and 1% bovine serum albumin (BSA) to block nonspecific binding sites, then incubated at 4°C for 24 h in mouse monoclonal mCherry (1:1000; ClonTech) in 0.01 m PBS with 0.3% Triton X-100 and 1% BSA. On the second day, sections were washed in PBS with 0.1% Triton X-100 and incubated for 60 min with a biotinylated goat anti-mouse antibody (1:400; Dako) in PBS with 1% BSA, washed again, and transferred to an avidin–biotin complex for 1 h (1:250; Vector Laboratories) in PBS-Triton X-100. Finally, sections were washed and developed using diaminobenzidine (DAB), rinsed, mounted on pregelatinized glass slides, dried, and coverslipped in DePex (BDH Chemicals). Overview images were obtained by use of a light microscope.

Dual-labeling immunofluorescence was performed as described elsewhere (Apuschkin et al., 2015) using rabbit polyclonal TH (1:1000; ADH Diagnostic) and mouse monoclonal mCherry (1:500; ClonTech) with subsequent species-appropriate Alexa Fluor 488–conjugated and Alexa Fluor 568–conjugated goat secondary antibodies (1:500; Life Technologies). Sections were mounted and coverslipped using Prolong Gold antifade reagent (Life Technologies) and analyzed with a Carl Zeiss AxioScan microscope.

All mice used for behavioral studies were subjected to postexperimental validation of midbrain mCherry expression. Following brain immersion fixation in 4% PFA for 24 h, coronal sections (40 μm) from the midbrain (and striatum in the initial cohorts) were mounted and coverslipped using Prolong Gold anti-fade reagent to assess mCherry expression directly by epifluorescence microscopy (Zeiss). If VTA mCherry expression was not detected in both hemispheres of bilaterally injected DREADD mice, they were excluded from further analysis.

### Electrophysiology

Midbrain slices (300 µm) were obtained from mice expressing hM4Di or mCherry using a vibratome (Leica VT1200). For coronal sections, slicing was performed in a chilled solution of sodium substituted artificial cerebrospinal fluid (ACSF) containing (in mm) NMDG 125, KCl 2.5, NaHCO_3_ 26, MgCl_2_ 3, NaH_2_PO_4_ 1.25, and glucose 25. For horizontal sections, slicing was performed in a chilled sucrose-based ACSF solution containing (in mm) sucrose 75, NaCl 67, NaHCO_3_ 26, glucose 25, KCl 2.5, NaH_2_PO_4_ 1.25, CaCl_2_ 0.5, and MgCl_2_ 7. Slices were transferred after slicing to 35°C ACSF containing (in mm) NaCl 125, KCl 2.5, NaHCO_3_ 26, CaCl_2_ 2, MgCl_2_ 1, NaH_2_PO_4_ 1.25, and glucose 25. The ACSF was continuously bubbled with 95% O_2_ and 5% CO_2_, and left for at least 1 h before measurement.

### Patch-clamp recording

Visual patch-clamp recordings of hM4Di-mCherry- and control mCherry-positive VTA neurons were performed with an upright microscope (Olympus BX51WI). The submerged recording chamber was continuously perfused with oxygenated ACSF. Glass pipettes were pulled on a puller (Sutter Instruments P87). Coronal sections were used for CNO puff application experiments, for which the pipette contained (in mm) K-gluconate 122, Na_2_-ATP 5, MgCl_2_ 2.5, CaCl_2_ 0.0003, Mg-Gluconate 5.6, K-Hepes 5, H-Hepes 5, EGTA 1, Biocytin 10 (Invitrogen), and Alexa Fluor 488 hydrazide 1. These recordings were performed in the presence of CNQX (AMPA receptor blocker), gabazin (GABA-A channel inhibitor), and DL-AP5 (NMDA receptor blocker) to silence synaptic activity and isolate the postsynaptic effects of hM4Di-mediated inhibition by local puff application of CNO (30 µm). Control recordings in slices from non-DREADD-expressing mCherry mice were performed in ACSF without inhibitors. Horizontal sections were used for AMPAR/NMDAR recordings, and the recording pipette contained (in mm) Cs-Gluconate 130, NaCl 8, MgCl_2_ 2.0, EGTA 2, CsCl 6, Hepes 6, QX-314 bromide 5, Na_2_-ATP_3_ 2.5, and Na_2_-GTP_3_ 0.5. The pH was adjusted to 7.3–7.4. Recordings were performed in whole-cell configuration. The recording electrodes (resistance 4–6 MΩ) were mounted on 3-axis motorized micromanipulators (Luigs and Neumann) and connected to CV-7B Current-Clamp and Voltage-Clamp Headstages (Molecular Devices). Recordings were acquired with a Multiclamp 700B amplifier and 1440A Digitizer (Molecular Devices). For AMPAR/NMDAR ratio recordings, picrotoxin was added to the ACSF. The cells were initially held at –70 mV, before being depolarized at 40 mV. Synaptic currents were evoked by stimuli (0.1 ms) at 0.067 Hz through bipolar stainless-steel electrodes positioned rostrally to the VTA. If series resistance (measured by –5-mV test pulse) varied by >25%, the experiment was terminated. The AMPAR/NMDAR ratio was computed as the averaged EPSC (14 traces) before and after application of DL-AP5 for minimum 5 min. AMPAR:NMDAR ratio was calculated by dividing the peak AMPAR-mediated EPSCs by the peak NMDAR-mediated EPSCs (calculated as the difference between the EPSC measured in the absence and presence of DL-AP5).

### Behavioral assays

Behavioral experiments were conducted during the light cycle. Mice were habituated to the test room at least 1 h before initiation of all experiments. For chemogenetic interventions, CNO (2 mg/kg) was administered i.p. 30 min before the test or the subsequent cocaine injection to ensure an adequate time span for the DREADDs to manipulate VTA DA activity. The DREADD experiments were conducted on cohorts of surgerized mice (*n* = 16–32), and data were pooled from 2–3 replicate experiments for habituation, acute response to cocaine, and behavioral sensitization to cocaine. These experiments were conducted in the order mentioned on the same mice. From one cohort, the saline control mice were used for the long-term CNO effect study. Electrophysiology recordings, projection-specific acute cocaine response, cocaine response following repeated cocaine in home cage, conditioned place preference, and reward/motivation assays were conducted in individual groups of mice in separate experiments.

### Habituation and acute response to cocaine in an open field test

Mice were placed in the center of square white open arenas (40 × 40 × 40 cm) and monitored for 90 min (novelty-induced locomotion and habituation) or 180 min (acute cocaine-induced locomotion). Open-field locomotion was recorded and analyzed using video-tracking software (Noldus), and distance traveled was divided into 5- or 10-min bins. In the novelty-induced locomotion and habituation tests, mice were administered CNO or VEH in their home cage 30 min before the test. On the following day, in the acute cocaine-induced locomotor test, mice were placed in the center of the arena and allowed to habituate for 90 min before CNO was administered. After another 30 min, the mice were given an injection of cocaine (20 mg/kg) or saline (0.9%), and locomotion was tracked for an additional 60 min to assess cocaine-induced locomotion. In a separate cohort of mice, these open field tests were conducted after repeated treatment with cocaine in the home cage. For 6 consecutive days, hM4Di and control mice were administered cocaine (20 mg/kg) at 10 am in their home cage. One week after the last injection, the mice were tested in the open field test as described above.

### Behavioral sensitization to cocaine and chronic CNO effects in activity chamber tests

One week after the acute experiment, the mice undertook a behavioral sensitization paradigm. This was conducted in novel activity boxes (30 × 30 × 20 cm) fitted with a beam-break movement-detection system (Med Associates). Mice were injected in their home cage with CNO or VEH 30 min before injection of cocaine (20 mg/kg) or saline, after which they were placed in the activity box, and locomotor activity was detected for 60 min. This was repeated for 6 consecutive days. After 14 d of withdrawal, the mice received a challenge dose of cocaine (20 mg/kg) without pretreatment. In a second challenge 1 wk later, mice were pretreated with CNO 30 min before a challenge dose of cocaine. On this day, the hM4Di saline control mice were not injected but placed in the activity box and tracked for 1 h as previously, as a baseline measure for chronic CNO test. The following 14 d, these mice received one daily (∼9 am) i.p. injection of CNO or VEH (i.e., the same as during induction). The day after the last injection, mice again were placed in the activity box and locomotor activity was measured.

### Cocaine conditioned place preference (CPP): setup and schedule

CPP experiments were performed in Med Associates square activity boxes fitted with beam-break movement detectors. An overview of CPP experimental setup and schedule is displayed in Fig. 7*A*. A split wall of red Plexiglas separated the chamber into two compartments (27.3 × 13.5 cm) to generate a forced-choice procedure with a predictable-preference design. One compartment, made of white decorated walls, a white smooth floor, and a transparent plastic lid was lighter than the other (lux: ∼50). The other, darker compartment (lux: ∼20) contained walls decorated with horizontal black and gray stripes, gray Lego-floor with the bottom side up, and a black plastic lid. During conditioning, compartments were fully separated by the split wall. However, on test days, the split wall contained a small hole (4 × 4 cm) to allow free passage between the two compartments. Time in each compartment was recorded, and the red split-walls allowed beam-break movement detection throughout the test session to track and analyze locomotor activity as well.

#### Pretest (day 1–3)

Baseline preference was determined from 3 days of pretesting before conditioning. On each day, the mice were placed in the light compartment and allowed to freely move between the light and dark compartment for a total of 20 min. Time spent in each compartment was recorded on each day, and all mice demonstrated a strong preference for the dark compartment. Therefore, cocaine was always paired with the less preferred, light compartment. Individual pretest preferences were relatively stable over the three test sessions, and the average was used to define baseline preference and groups. During conditioning sessions, mice were pretreated with VEH or CNO 30 min before cocaine (on conditioning sessions in the least preferred compartment) and before saline (on conditioning sessions in the preferred compartment). Control groups received saline in both compartments; one group pretreated with VEH, another with CNO, and a third group pretreated with VEH in the preferred and CNO in the least preferred compartment (see Fig. 7*B*).

#### Place-conditioning (day 4–11)

During the 8 d of conditioning, each mouse was injected with either saline or cocaine (10 mg/kg i.p.) immediately before placement in the appropriate compartment. To test the effect of CNO in hM4Di mice, CNO or VEH was injected 30 min before the cocaine/saline injection in the home cage. Activity levels were recorded throughout the 30-min session in which the mice were restricted to the appropriate compartment. Conditioning days were alternated so that mice were placed in a different compartment each day, but the compartments paired with cocaine, and the days on which they were exposed to this compartment, were counterbalanced.

#### CPP test (day 12)

On the day after the last conditioning session, postconditioning preferences were determined. Mice were again allowed to freely move between the two compartments in a 20-min test session where activity and time spent in each compartment was recorded.

#### Extinction (day 15–22)

Following establishment of CPP, the mice were daily placed in the CPP apparatus without any injections and allowed to freely move between the two compartments for 20 min. Loss of preference for the cocaine-paired compartment was followed throughout extinction. The last test was defined as the day when all mice were no different from the baseline preconditioning test for two consecutive days.

#### Reinstatement (10 d after last extinction)

Following a withdrawal phase lasting 10 d from the last extinction test, all mice received a priming dose of cocaine (5 mg/kg i.p.) before being placed in the light compartment and allowed to freely move between the two compartments for 20 min.

### Reward preference test (RPT)

The RPT was conducted as described elsewhere to assess perception of reward (Humby et al., 2005; Runegaard et al., 2017). Mice expressing hM4Di, food-deprived to 85%–90% of normal body weight, were exposed to water and sweetened milk (Mathilde strawberry milk, Arla Foods) for 20 min over the course of 3 consecutive days in 2 separate weeks. Drug treatment took place on the third day of each week, when mice were injected with VEH or CNO 30 min before the test. Drug treatment was counterbalanced, and mice were randomly allocated to a treatment group with 7 d between treatments.

### Must touch: 5-choice serial reaction time task, must-touch stage

Food-restricted mice were trained in modified nine-hole operant touchscreen-based chambers (40 × 34 × 42 cm; Campden Instruments) to reach high baseline performance at a 1.5-s stimulus duration probe in the 5-CSRTT as described elsewhere (Caballero-Puntiverio et al., 2017; Fitzpatrick et al., 2017). Then, mice were injected with hM4Di and allowed to recover for 3 wk before motivational aspects of reward were assessed in the must-touch stage. A lit square on the screen had to be touched to receive a reward (7 µl strawberry milk), which was collected at the reward tray and counted as a correct touch trial. A new trial was initiated once the mouse left the reward tray. This challenge took place for 20 min over the course of 3 consecutive days in 2 separate weeks, with drug treatment taking place on the third day. Number of touch trials completed and mean correct and reward collection latencies were recorded during each test session.

### High-performance liquid chromatography analysis

Striatal lysates for high-performance liquid chromatography (HPLC) analysis of DA content were prepared from hM4Di mice in the chronic CNO experiment. Following the locomotor activity test, mice were sacrificed, and following rapid dissection of the brain, NAc and dorsal striatum (dStr) were punched out from coronal slices. Tissue samples were immediately frozen on dry ice and stored at –80°C until homogenized in 500 μl perchloric acid 0.1 N. Following centrifugation at 14,000 × *g* for 30 min, 200 µl of the supernatant was filtered through a glass 0.22-µm filter (Avantec 13CP020AS). The samples were analyzed using EC-HPLC methodology. The concentrations of monoamines and metabolites were determined by HPLC with electrochemical detection. The column was a Prodigy 3 µm ODS-3 C18 (DA 2 × 100 mm, particle size 3 µm, Phenomenex, YMC Europe). The mobile phase (55 mm sodium acetate, 1 mm octanesulfonic acid, 0.1 mm Na_2_EDTA, and 8% acetonitrile, pH 3.2) was degassed with an online degasser. Samples (10 µl) were injected with a flow rate of 0.15 ml/min. The electrochemical detection was accomplished using an amperometric detector (Antec Decade) with a glassy carbon electrode set at 0.8 V and an Ag/AgCl reference electrode. The output was recorded and peak areas calculated by LC solution software (Shimadzu).

### Immunoblotting

Dorsal striatum (DStr) was dissected from coronal slices using a brain matrix and a puncher. Striata were homogenized in lysis buffer (1% Triton X-100, 0.1% SDS, 1 mm EDTA, 50 mm NaCl, 20 mm Tris, pH 7.5) with added inhibitor cocktail of proteases (cOmplete Protease Inhibitor Cocktail, Roche Diagnostics) and phosphatases (Phosphatase Inhibitor Cocktail 3, Sigma). Tissue preparations were mechanically disrupted using a motor-driven pestle (800 rpm) and mixed by turning them upside down and rotating at 4°C for at least 10 min. Homogenates were centrifuged at 16,000 × *g* for 10 min at 4°C to remove debris. Lysates were prepared for immunoblotting following protein determination by a standard BCATM Protein Assay kit (Thermo Scientific Pierce). Equal amounts of striatal samples were separated by SDS–polyacrylamide gel electrophoresis (any kDa gels, Bio-Rad) and transferred to Immobilon-P membranes (Merck Millipore). The membranes were blocked in PBS containing 0.05% Tween 20 and 5% dry milk and incubated overnight with antibodies against DAT (Millipore, MAB369, 1:1000). For detection of pTH, blocking was performed in PBS containing 0.05% Tween 20 with 5% polyvinylpyrrolidone-40 (Sigma-Aldrich), and incubated overnight with antibody against pTH (Cell Signaling, 1:1000). Following incubation with HRP-conjugated anti-rat or anti-rabbit antibodies (Thermo Scientific Pierce, 1:2000), the blots were visualized by chemiluminescence (GE Healthcare ECL-kit, GE Health care Life Sciences) using AlphaEase (Alpha Innotech) and quantified. To verify equal protein loading, the membranes were reprobed with an HRP-conjugated antibody against β-actin (Sigma, 1:10,000) to which protein levels of DAT and pTH were normalized. Band intensities were quantified using ImageJ software (v.1.48, NIH).

### Experimental design and statistical analyses

Prism software was used for statistical analysis of molecular and behavioral data with less complex designs (GraphPad Prism 6). Data were analyzed using unpaired two-tailed *t*-tests, one-way ANOVA, or repeated-measures two-way ANOVA, with Bonferroni’s *post hoc* test wherever appropriate. For behavioral data with more than two independent variables, statistical comparisons were conducted using InVivoStat (http://invivostat.co.uk), and data were analyzed using multifactorial ANOVAs as appropriate. Significant main effects and interactions were followed by the relevant pairwise comparisons using the planned comparison procedure. Data were log-transformed whenever required to stabilize the variance and help satisfy the parametric assumptions of the analysis. Data are presented as mean + SEM alongside individual data points in bar graphs wherever appropriate. Signiﬁcance level was set at *p* < 0.05. Plots were constructed using GraphPad Prism 6, and figures were created using Illustrator (Adobe Systems).

## Results

### Chemogenetic control of VTA DA neurons

**Figure 1.**
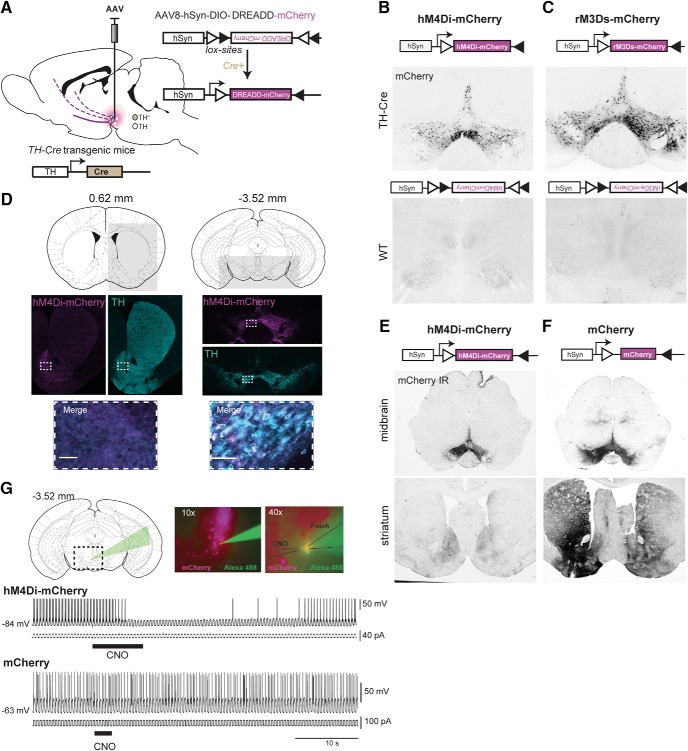
Targeting and modulating VTA dopaminergic signaling with DREADDs. ***A***, Injection of Cre-dependent DREADD virus (AAV-hSyn-DIO-hM4Di-mCherry or AAV-hSyn-DIO-rM3Ds-mCherry) in the midbrain of TH-Cre mice results in expression of the DREADD transgene in TH-positive VTA neurons and is distributed along their projections to target regions including ventral striatum. ***B***, ***C***, Cre-mediated control of expression demonstrated by representative images of mCherry fluorescence in midbrain slices from TH-Cre (upper panels) and WT (lower panels) mice injected with AAV carrying Cre-dependent hM4Di-mCherry (B) or rM3Ds-mCherry (C). ***D***, Schematics of coronal sections and area indicated by the light gray square of striatum (left) and midbrain (right), for which immunofluorescence of TH (turquoise) and hM4Di-mCherry (magenta) is shown individually below, together with a merged zoom of the dashed rectangular areas. Scale bar = 50 μm (representative sections, *n* = 9). Numbers over schematic sections indicate distance from bregma. ***E***, ***F***, Representative immunohistochemical images of mCherry in midbrain (upper panels) and striatum (lower panels) of TH-Cre mice injected with Cre-dependent hM4Di-mCherry (***E***) or mCherry (***F***) in the midbrain (as shown in ***A***) demonstrate that transgene translocation and expression pattern depend on the transgene. ***G***, Inhibition of action potentials in VTA DA neurons is accomplished only in slices expressing hM4Di, not mCherry only, following CNO application. Upper panel: Coronal section of midbrain slice with dashed rectangle and green arrow indicating area of patch-recording which is shown in a 10×-magnification picture of VTA neurons expressing mCherry-tagged hM4Di (magenta), of which one is patched and shown in 40× magnification with the position of the patch and CNO puff pipette. Lower panel: Upper trace, representative current clamp recording from a hM4Di-expressing neuron demonstrating transient inhibition of evoked action potentials following focal application of CNO (30 μM); lower trace, CNO (30 μm) application had no effect on an mCherry-expressing VTA neuron.

Stereotaxic injection of AAvs encoding Cre-dependent DREADDs hM4Di-mCherry or rM3Ds-mCherry in the midbrain of TH-Cre mice (Fig. 1*A*) induced robust expression of mCherry in the midbrain. VTA was stably targeted, with similar expression patterns observed for hM4Di- and rM3Ds-mCherry, while no mCherry expression was detected in Cre-negative (WT) littermates subjected to identical stereotaxic surgery (Fig. 1*B*,*C*). The mCherry expression was confined to TH-positive neurons within VTA and, although the substantia nigra (SN) was slightly infiltrated in some instances, it distributed mainly along the mesolimbic pathway, with axonal mCherry expression primarily seen in ventral striatum (Fig. 1*D*; only hM4Di is shown). In TH-Cre mice, when mCherry was not part of a fusion DREADD transgene, we observed wider expression of mCherry in striatum, probably reflecting uneven compartmentalization between these transgenes, as the somatodendritic expression pattern was similar (Fig. 1*E*,*F*). We confirmed functional DREADD expression by whole-cell recordings of hM4Di-expressing VTA neurons in coronal slices. In current clamp mode, hM4Di-expressing neurons demonstrated transient inhibition of evoked action potentials on CNO application, consistent with activation of an inhibitory Gi-pathway (Fig. 1*G* upper trace; *n* = 3). Importantly, no effect of CNO was observed in control mCherry-expressing neurons (Fig. 1*G* lower trace; *n* = 6).

### Bidirectional control of novelty-induced locomotor activity by chemogenetic regulation of Gi- and Gs-coupled signaling in VTA DA neurons

**Figure 2.**
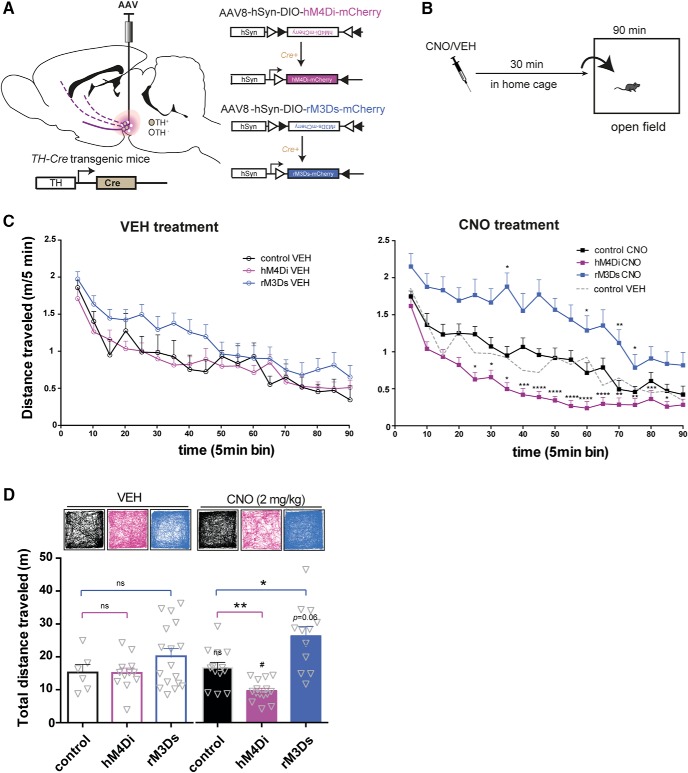
Bidirectional effects on locomotor habituation by hM4Di and rM3Ds stimulation in VTA. ***A***, AAV carrying hM4Di or rM3Ds was injected into the VTA of TH-Cre or WT mice**. *B***, Timeline of the behavioral setup applied to assess habituation of novelty-induced locomotor activity. CNO or vehicle (VEH) was injected i.p. in the home cage 30 min before placement into the center of an open field where locomotor activity was recorded and tracked for 90 min. ***C***, Time course of the 90-min habituation of novelty-induced locomotor activity in an open field 30 min after VEH (left) or CNO (right) injections in control (black), hM4Di-(magenta), or rM3Ds-(blue) expressing mice. To ease visual inspection, VEH- and CNO-treated mice are shown in different graphs. To aid comparison, the control mice treated with VEH have been added to the graph showing CNO-treated mice as a dashed gray line. While no significant difference was found between WT, hM4Di, and rM3Ds mice treated with VEH, CNO demonstrates bidirectional control of novelty-induced exploratory activity during habituation in hM4Di and rM3Ds mice while leaving WT mice unaffected (*F*_TIME_(17,1173) = 47.95, *p* < 0.0001, *F*_GROUP_(2,69) = 12.18, *p* < 0.0001, *F*_TREATMENT_(1,69) = 0.50, *p =* 0.48, and *F*_GROUP × TREATMENT_(2,69) = 7.36, *p =* 0.0013, mixed-model approach with group and treatment as between-subjects factors and time as repeated measure, followed by pairwise comparisons using the planned comparison procedure; ****, *p* < 0.0001, ***, *p* < 0.001, **, *p* < 0.01, *, *p* < 0.05 relative to CNO control, *n*: 12 hM4Di VEH, 17 rM3Ds VEH, 6 control VEH, 15 hM4Di CNO, 12 rM3Ds CNO, 13 control CNO). ***D*,** Representative tracks and graphs of total distance traveled during the habituation. Analysis of the total distance traveled in VEH- and CNO-treated mice shows significant reduction and increase in novelty-induced exploration following CNO in hM4Di and rM3Ds mice, respectively, compared to WT controls (*F*_TREATMENT_(1,69) = 0.02, *p =* 0.89, *F*_GROUP_(2,69) = 14.93, *p* < 0.0001, *F*_TREATMENTxGROUP_(2,69) = 5.37, *p =* 0.0068, two-way ANOVA, followed by planned comparisons between-groups; *, *p* < 0.05, **, *p* < 0.01 relative to CNO control, and within-group; ^#^, *p* < 0.05 and *p =* 0.06 relative to VEH, *n*: 12 hM4Di VEH, 17 rM3Ds VEH, 6 control VEH, 15 hM4Di CNO, 12 rM3Ds CNO, 13 control CNO). Data are shown as mean + SEM.

We next assessed whether bidirectional chemogenetic modulation of VTA activity altered locomotor activity during habituation to a novel environment. Hemizygous TH-Cre and WT littermates were injected with Cre-dependent stimulatory (the Gs-coupled rM3Ds) or inhibitory (the Gi-coupled hM4Di) DREADDs in VTA (Fig. 2*A*), giving rise to the following groups; hM4Di (TH-Cre expressing hM4Di), rM3Ds (TH-Cre expressing rM3Ds) and control mice (WT with no DREADD expression). Locomotor activity was assessed during a 90-min open field session, initiated 30 min after i.p. administration of CNO (2 mg/kg) or vehicle (VEH) in the home cage (Fig. 2*B*). All groups of mice habituated to the open field as attested by a significant main effect of time in a mixed-model analysis (Fig 2*C*; *F*_TIME_(17,1173) = 47.95, *p* < 0.0001, *F*_TREATMENT_(1,69) = 0.50, *p =* 0.48, *F*_GROUP_(2,69) = 12.18, *p* < 0.0001, *F*_GROUP × TREATMENT_(2,69) = 7.36, *p =* 0.0013, *n*: 12 hM4Di VEH, 17 rM3Ds VEH, 6 control VEH, 15 hM4Di CNO, 12 rM3Ds CNO, 13 control CNO). Importantly, there was no difference between VEH-treated control mice (WT), hM4Di-expressing mice, and rM3Ds-expressing mice, and no difference was observed between VEH- and CNO-treated control mice (Fig. 2*C*), as supported by pairwise comparisons between these groups. However, pairwise comparisons revealed significantly increased locomotion for CNO-treated M3Ds-expressing mice and significantly decreased locomotion for CNO-treated hM4Di-expressing mice, respectively, compared to CNO-treated control mice in the 25- to 85-min time window (Fig 2*C*; ****, *p* < 0.0001, ***, *p* < 0.001, **, *p* < 0.01, *, *p* < 0.05 relative to control CNO). Accordingly, the total distance traveled during habituation (Fig. 2*D*) revealed no difference between VEH-treated control mice, hM4Di-expressing mice, and rM3Ds-expressing mice but was significantly and oppositely changed in CNO-treated M3Ds-expressing mice and CNO-treated hM4Di-expressing mice compared to CNO-treated control mice (Fig. 2*D*; *F*_TREATMENT_(1,69) = 0.02, *p =* 0.89, *F*_GROUP_(2,69) = 14.93, *p* < 0.0001, *F*_INTERACTION_(2,69) = 5.37, *p =* 0.0068, two-way ANOVA followed by pairwise comparisons between groups). In addition, within-group comparisons of treatment effects revealed that CNO reduced the total distance traveled in hM4Di-expressing mice (*p =* 0.0103) and tended to increase activity of rM3Ds-expressing mice (*p =* 0.0605). In contrast, CNO had no effect on control mice (WT) as there was no difference between these control mice treated with CNO and VEH (*p =* 0.68; Fig. 2*D*). These observations suggest that Gi- and Gs-coupled signaling in TH-positive VTA neurons can substantially and bidirectionally regulate novelty-induced exploratory activity and habituation to the open field.

### VTA inhibition by hM4Di attenuates the acute locomotor response to cocaine

**Figure 3.**
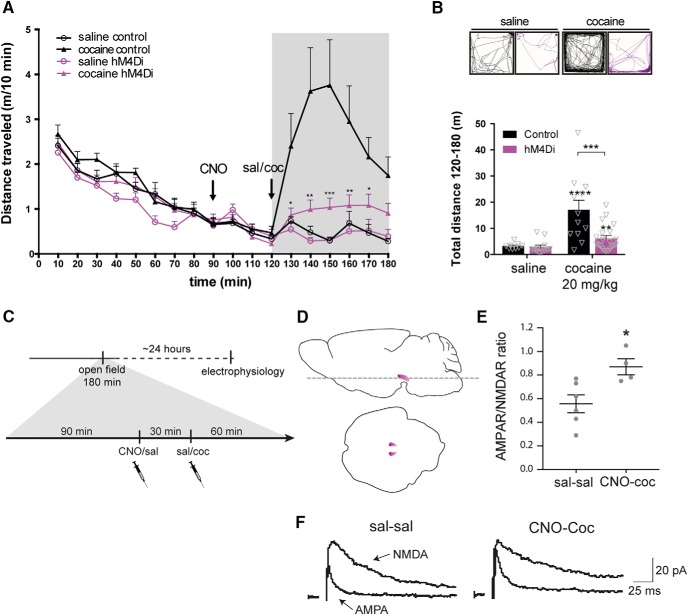
hM4Di-mediated inhibition inhibits the acute locomotor response to cocaine but does not prevent potentiation of VTA DA neurons following cocaine. ***A***, Acute cocaine-induced locomotor activity following habituation assessed in an open field in WT and TH-Cre mice injected with Cre-dependent hM4Di in the VTA. Distance traveled is shown as meters traveled per 10 min (m/10 min) and reveals significantly diminished cocaine-induced locomotor response following CNO treatment in TH-Cre mice expressing hM4Di compared to WT mice that did not express hM4Di (control; 120–180 min; *F*_TIME_(6,300) = 11.57, *p* < 0.0001, *F*_GROUP_(1,50) = 7.68, *p =* 0.0078 and *F*_TREATMENT_(1,50) = 36.17, *p* < 0.0001, mixed-model approach followed by planned comparisons; ***, *p* < 0.001, **, *p* < 0.01, *, *p* < 0.05 compared to cocaine control, *n*: 9 saline control, 12 cocaine control, 12 saline hM4Di, 21 cocaine hM4Di). ***B***, Representative tracks and graphs with total distance traveled after cocaine injection (120–180 min). Control mice showed clear cocaine-induced hyperlocomotor activity, which is impeded in hM4Di mice (*F*_GROUP_(1,50) = 9.71, *p =* 0.003, *F*_TREATMENT_(1,50) = 25, *p* < 0.0001, mixed-model approach followed by planned comparisons; ****, *p* < 0.0001 cocaine control versus saline control, **, *p =* 0.0097 cocaine hM4Di compared to saline hM4Di, ***, *p =* 0.0005 cocaine hM4Di compared to cocaine controls, *n*: 9 saline control, 12 cocaine control, 12 saline hM4Di, 21 cocaine hM4Di). ***C***, Timeline of experimental setup to assess the influence of hM4Di-mediated inhibition on the effect of cocaine on AMPAR/NMDAR in VTA DA neurons. As in ***A***, mice expressing hM4Di were placed in an open field and, after 90-min habituation, injected with CNO, 30 min before cocaine (20 mg/kg). Control mice were given saline injection. 60 min after cocaine injection, mice were placed back in home cage until the following day, when mice were sacrificed, and horizontal midbrain sections (as illustrated in ***D***) were prepared for electrophysiology. ***E***, Dot plot of individual AMPAR/NMDAR ratios 1 d after acute cocaine injection in hM4Di mice treated with CNO compared to hM4Di mice treated with saline. Stimulation of hM4Di did not prevent cocaine-induced potentiation of VTA DA neurons (*t*_8_ = 2.89, *p =* 0.02, unpaired *t* test, *n*: 6 sal-sal, 4 CNO-cocaine). ***F***, Representative traces of AMPA and NMDA currents. Data are mean ± SEM.

The suppressed novelty-induced locomotor activity on CNO stimulation of hM4Di in VTA neurons (Fig. 2*C*) was conceivably a result of reduced continuous release of DA. Since cocaine inhibits DA reuptake via DAT and thereby increases extracellular DA levels (Giros and Caron, 1993; Kristensen et al., 2011), we hypothesized that hM4Di-mediated inhibition would also impair the acute hyperlocomotor response to cocaine. The acute effect of cocaine (20 mg/kg) following hM4Di-mediated inhibition was assessed in the same open field used for the novelty-induced locomotor assay, with CNO-treated WT mice serving as controls. Hence, all groups were pretreated with CNO, assuming that CNO has no influence on locomotion in the control mice, as suggested by the initial habituation experiments (Fig. 2*C*). Importantly, TH-Cre and WT mice injected with hM4Di in VTA were allowed to habituate in the open field for 90 min before i.p. injection of CNO (2 mg/kg) 30 min before a saline or cocaine injection (Fig. 3*A*). Note, that during the habituation phase (0–90 min), all four groups showed similar locomotor activity (Fig. 3*A*, 0–90 min; *F*_TIME_(8400) = 86.89, *p* < 0.001, *F*_GROUP_(1,50) = 2.46, *p =* 0.123, *F*_TREATMENT_(1,50) = 2.06, *p =* 0.157, mixed-model approach, *n*: 9 saline control, 12 cocaine control, 12 saline hM4Di, 21 cocaine hM4Di). While WT mice (controls) exhibited an expected hyperlocomotor response to cocaine after CNO pretreatment, the behavioral response to cocaine was markedly diminished in TH-Cre mice expressing hM4Di (Fig. 3*A*, 120–180 min; *F*_TIME_(6,300) = 11.57, *p* < 0.0001, *F*_GROUP_(1,50) = 7.68, *p =* 0.0078 and *F*_TREATMENT_(1,50) = 36.17, *p* < 0.0001, mixed-model approach followed by planned comparisons; ***, *p* < 0.001, **, *p* < 0.01, *, *p* < 0.05 compared to cocaine control, *n*: 9 saline control, 12 cocaine control, 12 saline hM4Di, 21 cocaine hM4Di). Analysis of the total distance traveled after cocaine treatment (120–180 min) revealed an overall main effect of group (Fig. 3*B*, F_GROUP_(1,50) = 9.71, *p =* 0.003) and treatment (Fig. 3*B*, F_TREATMENT_(1,50) = 25, *p* < 0.0001). Planned comparisons showed that although cocaine induced a hyperlocomotor response after CNO pretreatment in both controls (****, *p* < 0.0001 compared to saline control) and hM4Di-expressing mice (**, *p =* 0.0097 compared to saline hM4Di), the cocaine-induced locomotor response was significantly impaired in hM4Di mice (***, *p =* 0.0005 compared to cocaine controls; Fig. 3*B*). Interestingly, there was no difference between TH-Cre mice expressing hM4Di and WT control mice treated with saline after 90-min habituation and CNO administration (*p =* 0.3276). Hence, in contrast to novelty-induced explorative locomotor activity, basal locomotion was not affected by hM4Di-mediated inhibition (Fig. 3*A*,*B*). The data support the view that the behavioral response to cocaine relies on VTA DA neuronal activity and suggest that engagement of a Gi-coupled pathway in VTA DA neurons via hM4Di reduces neuronal activity and prevents cocaine-induced effects. Besides the acute behavioral hyperlocomotor response to cocaine, a single injection of cocaine has been associated with neuronal plasticity of VTA DA neurons manifested as an increased AMPAR/NMDAR ratio 24 h after cocaine exposure (Ungless et al., 2001). An important question is whether this effect of cocaine is prevented by hM4Di-mediated inhibition concomitant with inhibition of the locomotor response. Interestingly, electrophysiological analysis of acute midbrain slices from hM4Di-expressing mice 24 h after acute cocaine and CNO injections showed that this was not the case (Fig. 3*C*,*D*). Patch-clamp recordings of mCherry-positive neurons demonstrated significantly increased AMPAR/NMDAR ratio following CNO + cocaine compared to saline control treatment (Fig. 3*E*,*F*; *t*_8_ = 2.89, *p =* 0.02, unpaired *t* test, *n*: 6 sal-sal, 4 CNO-cocaine). Thus, cocaine-induced plasticity occurred despite attenuation of the acute locomotor response following CNO-mediated stimulation of hM4Di.

### Cocaine-induced locomotion is conveyed by VTA-NAc projecting DA neurons

**Figure 4.**
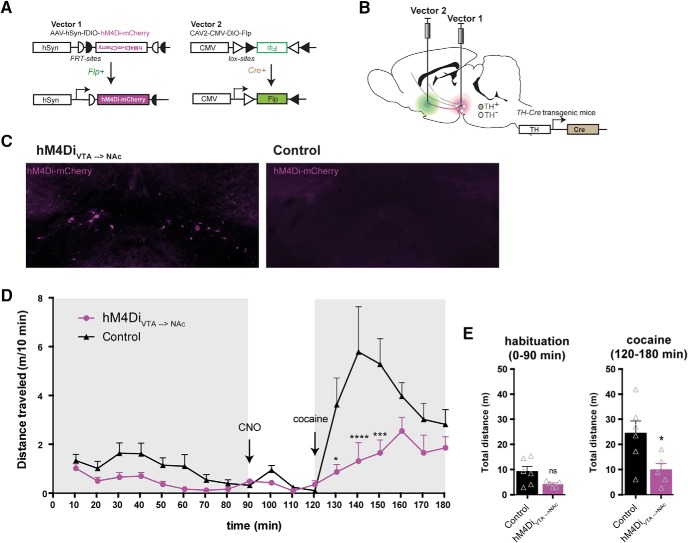
Stimulation of hM4Di selectively in VTA to NAc projecting DA neurons recapitulates the inhibitory effect on cocaine locomotion. ***A***, ***B***, The two viral vectors used in the dual-viral approach to specify expression of hM4Di to DA neurons projecting from VTA to NAc; the Flp-dependent DREADD virus, AAV-hSyn-fDIO-hM4Di-mCherry (Vector 1) was injected into the midbrain of TH-Cre mice and the Cre-dependent CAV2-DIO-Flp (Vector 2) was injected in the ventral striatum to specify hM4Di expression to TH-positive neurons projecting from VTA to the ventral striatum. ***C***, Representative immunofluorescent images of hM4Di-mCherry (magenta) in coronal midbrain sections of TH-Cre (hM4Di_VTA→NAc)_ and WT control. ***D***, Time course of open field session in which the acute response to cocaine (20 mg/kg) was assessed after 90-min habituation and 30 min with CNO (2 mg/kg), revealing a significant attenuation of the immediate locomotor response to cocaine (*F*_GROUP_(1,9) = 6.754, *p* = 0.0288, two-way repeated-measures ANOVA, *n*: 6 WT control, 5 hM4Di_VTA→NAc_. Bonferroni’s *post hoc* test; ****, *p* < 0.0001, *** *p* < 0.001, and *, *p* < 0.05 relative to WT control). ***E***, Total distance traveled during habituation (0–90 min; left) and after cocaine administration (120–180 min; right) demonstrates that while no significant difference between groups was revealed during habituation (*t*_9_ = 2.072, *p =* 0.0681, unpaired *t* test), activation of hM4Di specifically in the VTA neurons projecting to NAc is sufficient to dampen the cocaine locomotor response compared to WT controls (*t*_9_ = 2.439, *p =* 0.037, unpaired *t* test). Data are mean + SEM.

Another key question is whether the reduction of the acute cocaine locomotor response on hM4Di-mediated inhibition can be fully attributed to VTA DA neurons (as some degree of expression was found in SN) or even specific VTA projections. To address this, we adapted a dual-viral strategy based on application of retrogradely transported CAV2 (Schwarz et al., 2015) carrying a Cre-dependent Flp (DIO-Flp) transgene to restrict expression of a Flp-dependent hM4Di to dopaminergic neurons projecting from the VTA to NAc (Fig. 4*A*,*B*). The CAV2-DIO-Flp was injected into the ventral striatum, whereas the Flp-dependent AAV-fDIO-hM4Di-mCherry was injected into the VTA of TH-Cre and WT mice, resulting in hM4Di-mCherry expression in TH-Cre mice only (Fig. 4*C*). When hM4Di expression was specifically confined to TH-expressing neurons projecting toward the ventral striatum, we observed a similar reduction in cocaine-induced hyperlocomotion when CNO was pre-administered (Fig. 4*D*; *F*_TIME_(17,153) = 11.55, *p* < 0.0001, *F*_GROUP_(1,9) = 6.754, *p =* 0.0288, and *F*_INTERACTION_(17,153) = 3.455, *p* < 0.0001, two-way repeated-measures ANOVA, Bonferroni’s *post hoc* test; ****, *p* < 0.0001, *** *p* < 0.001, and *, *p* < 0.05 relative to WT control, *n*: 6 WT control, 5 hM4Di_VTA→NAc_). Note that although the hM4Di_VTA→NAc_ mice seemed to move slightly less during habituation, no significant difference between the groups was found before cocaine administration. In addition, assessment of the total distance traveled during habituation (0–90 min) revealed no significant difference (Fig. 4*E* left; *t*_9_ = 2.072, *p =* 0.0681, unpaired *t* test), while the total distance traveled following cocaine (120–180 min) was significantly reduced in hM4Di_VTA→NAc_ mice (Fig. 4*E* right; *t*_9_ = 2.439, *p =* 0.037, unpaired *t* test). This suggests that reduction of cocaine-induced locomotor behavior following VTA DA hM4Di-mediated inhibition was primarily conveyed by dopaminergic VTA-NAc projections.

### Assessment of repeated hM4Di-mediated inhibition of VTA DA neurons in a behavioral sensitization paradigm

**Figure 5.**
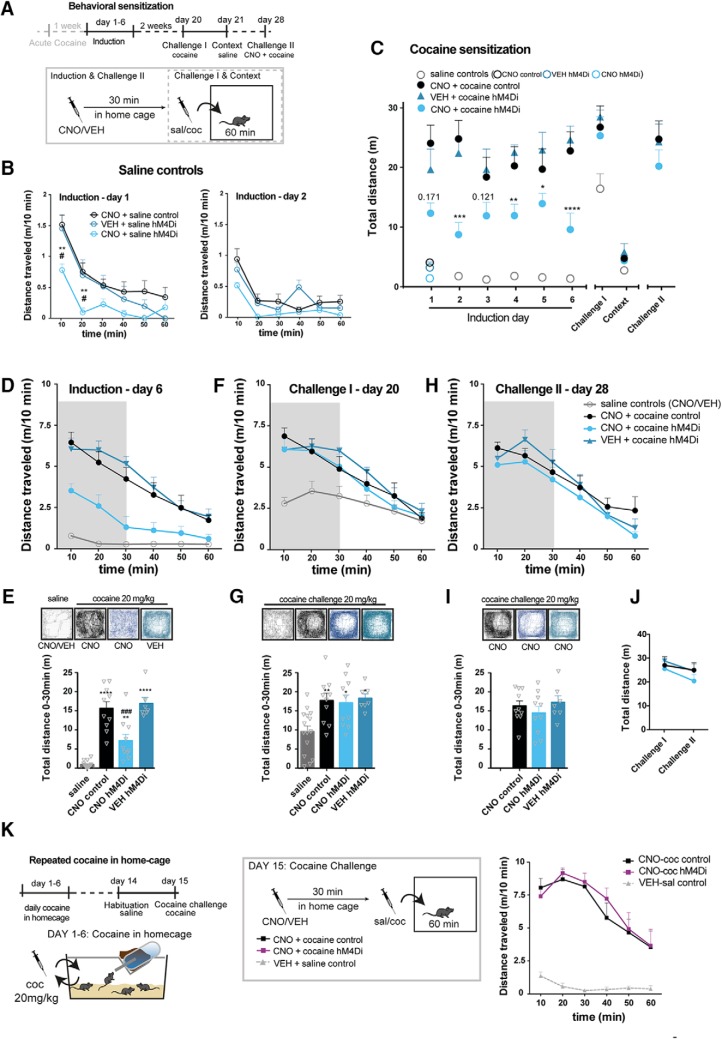
Repeated cocaine exposure diminishes the inhibitory effect of hM4Di on locomotor activity. ***A***, Timeline of behavioral sensitization paradigm conducted 1 wk after the acute cocaine experiment in the open field test. Locomotor activity was tracked for 60 min in activity boxes immediately after cocaine/saline administration on each day of induction (days 1–6) challenge- and context test days. During induction control (WT) or hM4Di (TH-Cre) mice were treated for six consecutive days with cocaine or saline 30 min after CNO or VEH pretreatment defining the following groups; saline controls, CNO + cocaine control (WT), CNO + cocaine hM4Di and VEH + cocaine hM4Di. Saline control mice were WT mice pretreated with CNO, hM4Di mice pretreated with CNO, or hM4Di mice pretreated with VEH. On the Challenge I and context test days, there were no pretreatments, and mice were placed in the activity boxes immediately after cocaine or saline injections. On the Challenge II test day, all mice were pretreated with CNO 30 min before the cocaine challenge. ***B***, Locomotor activity of saline-treated mice on the first days of induction, day 1 (left) and 2 (right), shown as meters traveled per 10 min (m/10 min). Only in the initial phase of habituation on induction day 1, CNO-treated hM4Di mice demonstrated reduced locomotion during habituation (induction day 1; *F*(2,12) = 4.688, *p =* 0.031, two-way repeated-measures ANOVA, Bonferroni’s *post hoc* test; **, *p* < 0.01 compared to CNO control, #, *p* < 0.05 compared to VEH hM4Di. Induction day 2; *F*(2,12) = 3.249, *p =* 0.075, two-way repeated-measures ANOVA, *n*: 7 CNO control, 4 VEH hM4Di, 4 CNO hM4Di). From induction day 2, there was no difference, and saline control groups were pooled for further analysis. ***C***, Total locomotor activity during induction, challenges, and context control days of cocaine and saline control groups (the latter shown as one group as from induction day 2). Locomotor activity after cocaine administration was reduced when hM4Di mice were pretreated with CNO during induction (*F*_GROUP_(3,38) = 35.84, *p* < 0.0001, ****, *p* < 0.0001, ***, *p* < 0.001, **, *p* < 0.01 *, *p* < 0.05 compared to VEH + cocaine hM4Di, Bonferroni’s *post hoc* tests after significant two-way repeated-measures ANOVA, *n*: 15 saline controls (pooled 7 controls and 4 hM4Di pretreated with CNO and 4 hM4Di pretreated with VEH) and the following cocaine groups: 10 CNO control, 10 CNO hM4Di, 7 VEH hM4Di). ***D***, Locomotor activity on the last day of induction, day 6, shown as m/10 min. ***E***, Representative tracks and graphs of total distance traveled during the initial 30 min of the test shown in ***D***. On the last day of induction, CNO-treated hM4Di mice moved significantly less than CNO-treated controls and VEH treated hM4Di mice (*F*(3,39) = 37.22, *p* < 0.0001, one-way ANOVA. Bonferroni’s *post hoc* test; **** *p* < 0.0001, **, *p* < 0.01 relative to saline control, ^###^, *p* < 0.001 relative to cocaine CNO control). Likewise, locomotor activity and representative tracks are shown for challenge I (in ***F*** and ***G***), where the mice received a challenge-dose of cocaine 20 mg/kg immediately before testing and for challenge II (in ***H*** and ***I***), where all mice were pretreated with CNO (2 mg/kg) 30 min before the cocaine challenge. ***G***, On Challenge I, all mice treated with cocaine during induction demonstrated significant increased locomotor activity to the challenge dose compared to mice that during induction were treated with saline (*F*(3,38) = 6.385, *p =* 0.0013, one-way ANOVA, Bonferroni’s *post hoc* test; **, *p* < 0.01, *, *p* < 0.05 relative to saline control). ***I***, hM4Di stimulation had no effect on locomotor activity in sensitized mice on a challenge dose of cocaine, as there was no difference in the cocaine-induced response between groups (*F*_GROUP_(2,24) = 0.7356, *p =* 0.4897, one-way ANOVA, or in the response between challenge I and challenge II as shown in ***J***. ***J***, Total distance traveled during the 60 min of each challenge (*F*_TIME_(1,24) = 4.145, *p =* 0.053, two-way repeated-measures ANOVA. ***K***, Left: timeline of repeated cocaine administration in home cage and subsequent assessment of CNO effects on cocaine-induced locomotion in a cocaine challenge. Middle: groups and experimental setup of the cocaine challenge. Right: cocaine induced locomotor activity of CNO pretreated hM4Di and control mice that have received repeated cocaine injections in home cage (6 daily injections, the last given a week before the test), shown as m/10 min. The cocaine-induced hyperlocomotor response in an open field was unaltered by CNO in hM4Di-expressing mice when they have been treated repeatedly with cocaine in an unrelated environment (home cage), with no difference between hM4Di and control mice treated with cocaine (*F*_GROUP_(1,8) = 0.1655, *p =* 0.6948, two-way repeated-measures ANOVA). Data are shown as mean + SEM.

We next asked whether inhibition of the behavioral response to acute cocaine following hM4Di-mediated inhibition would translate into an inhibition of behavioral sensitization on repeated cocaine exposure. Such a sensitization paradigm is thought to reflect the neural adaptations that contribute to and possibly gate the transition to addiction (Kalivas et al., 1998; Steketee and Kalivas, 2011). One week after the initial cocaine exposure, the same control and hM4Di mice were subjected to repeated cocaine administration as part of a behavioral sensitization protocol. In this experiment, mice were pretreated with either VEH or CNO in their home cage 30 min before the cocaine injection that was given immediately before the mice were placed in activity boxes and monitored for 60 min (Fig. 5*A*). A group of mice served as saline controls and included hM4Di-injected TH-Cre and WT mice. These mice were repeatedly administered saline following pretreatment with VEH or CNO and confirmed a CNO-specific reduction of locomotion during habituation to a novel environment in hM4Di-expressing mice (Fig. 5*B*, induction day 1; *F*(2,12) = 4.688, *p =* 0.031, two-way repeated-measures ANOVA, Bonferroni’s *post hoc* test; **, *p* < 0.01 compared to CNO control, #, *p* < 0.05 compared to VEH hM4Di.). Already on the second day, novelty-induced locomotion seemed reduced and CNO appeared not to further reduce locomotion during habituation in hM4Di-expressing mice (Fig. 5*B*, induction day 2; *F*(2,12) = 3.249, *p =* 0.075, two-way repeated-measures ANOVA, *n*: 7 CNO control, 4 VEH hM4Di, 4 CNO hM4Di). These observations support that the effects of CNO on locomotion are specific for hM4Di-expressing mice and suggest that hM4Di-mediated inhibition of VTA DA neurons does not influence basal locomotion. Therefore, these saline controls were pooled after induction day 1, after which there were no differences between groups (Fig. 5*C*).

### hM4Di-mediated inhibition of VTA DA neurons does not prevent behavioral sensitization to cocaine

To address specifically whether hM4Di-mediated inhibition of VTA DA neurons influenced cocaine-induced locomotion on repeated cocaine exposures, hM4Di-expressing mice were pretreated with CNO, and the cocaine-induced locomotor activity was compared to that of hM4Di-expressing mice pretreated with VEH and WT control mice pretreated with CNO (Fig. 5*C*). First, when comparing to the saline mice, the locomotor activity was clearly increased in the cocaine groups, but we noticed that none of the groups showed a gradual sensitization response to cocaine during the induction phase, which possibly was a consequence of the previous exposure to cocaine. Nonetheless, with daily repeated administration of cocaine (induction days 1–6), both control and VEH pretreated hM4Di-expressing mice exhibited a clear and alike cocaine-induced locomotor response, while locomotor activity was recurrently attenuated by CNO in hM4Di-expressing mice (Fig. 5*C*; *F*_TIME_(8,304) = 38.71, *p* < 0.0001, *F*_GROUP_(3,38) = 35.84, *p* < 0.0001, and *F*_INTERACTION_(24,304) = 5.546, *p* < 0.0001, two-way repeated-measures ANOVA, Bonferroni’s *post hoc* test, ****, *p* < 0.0001, ***, *p* <0.001, **, *p* <0.01, *, *p* <0.05 compared to VEH + cocaine, *n*: 15 saline controls (pooled 7 controls and 4 hM4Di pretreated with CNO and 4 hM4Di pretreated with VEH) and the following cocaine groups: 10 CNO control, 10 CNO hM4Di, 7 VEH hM4Di). In contrast to the significant impact of hM4Di-mediated inhibition on the acute cocaine response (Figs. 3*A*,*B* and 4*D*,*E* ) as well as on 6 consecutive days during the induction phase (Fig. 5*C*and locomotor activity during Induction day 6 shown in Fig. 5*D*,*E*, with the total distance traveled shown and analyzed in Fig. 5*E*; *F*(3,38) = 36.06, *p* < 0.0001, one-way ANOVA, Bonferroni’s *post hoc* test; ****, *p* < 0.0001, **, *p* < 0.01 relative to saline control, ###, *p* < 0.001 relative to cocaine CNO control and cocaine VEH hM4Di), 14 d after the last cocaine injection, there was no effect of hM4Di-mediated inhibition on the expression of behavioral sensitization as determined by a challenge dose of cocaine (20 mg/kg; Challenge I; Fig. 5*C*,*F*,*G* with total distance traveled shown and analyzed in Fig. 5*G*; *F*(3,38) = 6.385, *p =* 0.0013, one-way ANOVA, Bonferroni’s *post hoc* test; **, *p* < 0.01, *, *p* < 0.05 relative to saline control). Instead, the response to cocaine was similar to that observed in CNO pretreated WT control mice and in VEH pretreated hM4Di-expressing mice (Fig. 5*F*,*G*).

### Attenuation of cocaine-induced locomotion by hM4Di signaling is suppressed following repeated cocaine exposures

In a second challenge 1 wk later (day 28), mice received an injection of CNO before cocaine. Surprisingly, CNO did not inhibit the locomotor response to cocaine in either hM4Di-expressing mice pretreated with CNO or CNO naive hM4Di-expressing mice that were pretreated with VEH during the induction phase (Fig. 5*C*,*H*,*I*, with total distance traveled shown and analyzed in Fig. 5*I*; *F*_GROUP_(2,24) = 0.7356, *p =* 0.4897, one-way ANOVA, and Fig. 5*J*; *F*_TIME_(1,24) = 4.145, *p =* 0.053, two-way repeated-measures ANOVA). To further address the lack of effect of hM4Di after repeated cocaine exposures, another cocaine experiment was conducted in mice treated repeatedly with cocaine in their home cages (1 daily injection for 6 consecutive days, with last injection administered 1 week before the open field test). Again, no difference was observed between these groups of mice (Fig 5*K*; *F*_TIME_(5,40) = 13.59, *p* < 0.0001, *F*_GROUP_(1,8) = 0.1655, *p =* 0.6948, and *F*_INTERACTION_(5,40) = 0.3572, *p =* 0.8746, two-way repeated-measures ANOVA). Together, these experiments suggest that metabotropic signaling of hM4Di in VTA DA neurons can suppress both acute and early consecutive responses to cocaine, but after more chronic cocaine exposures, the sensitivity of this signaling is disrupted.

### Repeated inhibition of VTA DA neurons by hM4Di does not affect DA levels and locomotor behavior

**Figure 6.**
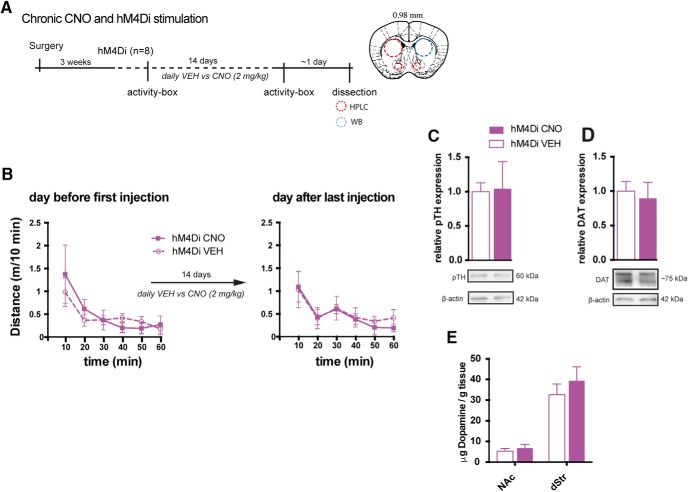
Chronic CNO administration does not alter basal behavior and DA homeostasis. ***A***, Timeline of the experimental setup to assess behavioral and cellular effects of repeated CNO administration in TH-Cre mice expressing hM4Di within the DA system. Locomotor activity was assessed in activity boxes before and after 14 days of daily CNO or VEH injections. Subsequently, brains were dissected and striatal areas subject to HPLC and WB assays to assess DA homeostatic parameters. ***B***, Locomotion in activity boxes the day before first injection, and the day after the last injection of 14-days repeated injections of VEH or CNO in mice expressing hM4Di in VTA DA neurons. Repeated stimulation of hM4Di did not induce adaptations affecting locomotion in the activity box the day after last injection (*F*_GROUP_(1,6) = 0.1646, P = 0.699 by two-way repeated-measures ANOVA, *n* = 4). ***C–E***, The brains were dissected, and molecular homeostasis of DA was assayed. There was no change in protein levels of pTH (***C***) and DAT (***D***) in dStr as assessed by WB (pTH: *t*(6) = 0.1014, *p =* 0.9225; DAT: *t*(6) = 0.3857, *p =* 0.7130, unpaired *t* test, *n* = 4), nor in DA levels of NAc and dStr (***E***) as assessed by HPLC (NAc: *t*(6) = 0.63, *p =* 0.547; dStr: *t*(6) = 0.807, *p =* 0.45, unpaired *t* test, *n* = 4). Data are shown as mean + SEM. Note, in this experiment, non-DREADD CNO controls were not included.

Repeated inhibition of VTA DA neurons could potentially cause aberrations in dopaminergic homeostasis and possibly affect our results. Therefore, DA homeostasis was assessed in hM4Di-expressing mice on the day after the last injection of 14 d with daily i.p. administration of CNO (2 mg/kg) or VEH (Fig. 6*A*). Furthermore, locomotor activity was assessed the day before and after the last injection of CNO or VEH and revealed no change in habituation to the activity boxes after chronic CNO treatment and hM4Di-mediated inhibition (Fig. 6*B*; *F*_TIME_(5,30) = 8.266, *p* < 0.0001, *F*_GROUP_(1,6) = 0.1646, *p =* 0.699, and *F*_INTERACTION_(5,30) = 0.3483, *p =* 0.8793, two-way repeated-measures ANOVA, *n* = 4). In the dissected brains, striatal levels of markers for DA homeostasis, including the phosphorylated (at serine residue 40) active form of TH (pTH; Lindgren et al., 2000) and DAT, were similar between CNO- and VEH-administered mice (Fig. 6*C*,*D*; pTH: *t*_6_ = 0.1014, P = 0.9225, and DAT: *t*_6_ = 0.3857, *p =* 0.7130, unpaired *t* test, *n* = 4). HPLC analysis did not reveal any changes in total levels of DA in dStr and NAc, further supporting intact DA homeostasis following chronic CNO (Fig. 6*E*; NAc: *t*_6_ = 0.63, *p =* 0.547, and dStr: *t*_6_ = 0.807, *p =* 0.45, unpaired *t* test, *n* = 4). Thus, repeated administration and daily activation of Gi-signaling in VTA neurons by 2 mg/kg CNO appears to have negligible influence on overall DA synthesis and turnover and does not alter DA-sensitive behaviors.

### hM4Di-mediated inhibition of VTA during conditioning does not prevent cocaine-induced place preference

**Figure 7.**
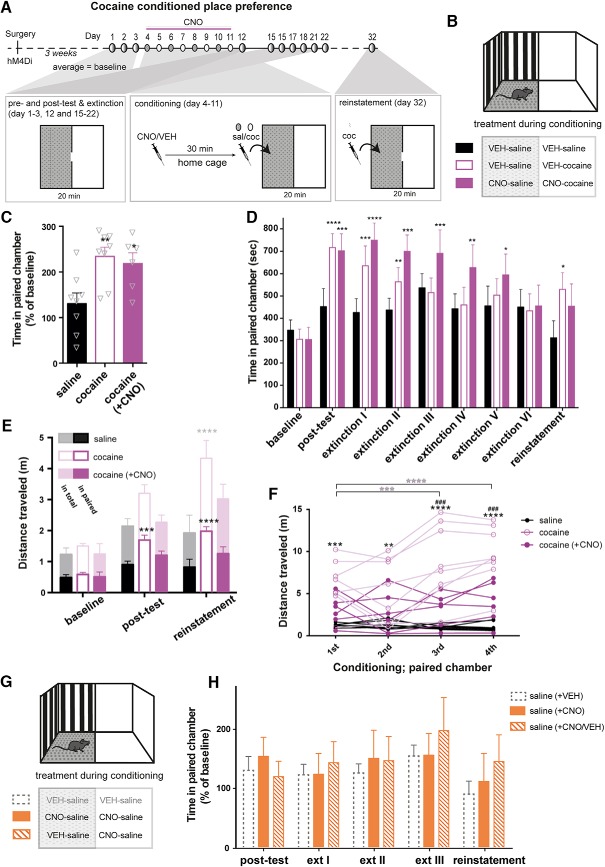
hM4Di-stimulation segregates hyperlocomotion from reward in a CPP paradigm. ***A***, Timeline and experimental protocol for cocaine-induced CPP in mice with hM4Di expression in VTA DA neurons. Below the timeline is an upside-down illustration of the biased two-compartment apparatus (shown in ***B***) and treatment during the various components of the CPP. During pretest, posttest, extinction, and reinstatement sessions, partitions with a small opening separated the light and dark compartments, which allowed mice to freely move between the two compartments during the 20-min sessions. During conditioning, mice were pretreated with CNO or VEH 30 min before injection of saline or cocaine, after which they were placed in and confined to one of the compartments for 30 min. Cocaine was always given in the least preferred (light) compartment, but treatment and sequence were alternated between groups. A 3-day pretest period provided a stable baseline for comparison. Eight days of conditioning was followed by a posttest and active extinction training session until the time spent in the paired compartment was not significantly different from baseline levels. Ten days after the last extinction day, the mice received a primed cocaine dose of 5 mg/kg to assess reinstatement. ***B***, Illustration of the CPP apparatus with a light (white walls and smooth floor) and a dark (black and gray striped walls and gray Lego-grip floor) compartment. Treatment during conditioning (in each compartment) for each of the three groups is shown below. ***C***, Time spent in cocaine-paired (light) compartment in percentage of baseline (average of pretests for each group) shows significant conditioning of both cocaine groups when compared to saline-treated mice (*F*(2,19) = 6.549, *p =* 0.0069, one-way ANOVA, *n*: 8 saline, 6 cocaine (+ CNO), 8 cocaine. Bonferroni’s *post hoc* test; **, *p* < 0.01, *, *p* < 0.05 relative to saline). ***D***, Time spent in the cocaine-paired compartment during baseline, posttest, extinction, and reinstatement demonstrates that while hM4Di-stimulation (i.e. CNO treatment) before cocaine during conditioning does not prevent cocaine-induced place preference, it prolongs the extinction phase, as these mice took more sessions before they lost their cocaine-induced place preference (*F*_INTERACTION_(16,152) = 1.833, *p =* 0.0315, *p* < 0.0001, ***, *p* < 0.001, **, *p* < 0.01 compared to baseline, Bonferroni’s *post hoc* test after significant two-way repeated-measures ANOVA). ***E***, Distance traveled in total (shadowed) and cocaine-paired (solid) compartment during the 20-min sessions of baseline, posttest, and reinstatement. hM4Di-stimulation before cocaine during conditioning prevents context-induced locomotor activity as well as sensitized response to a lower dose of cocaine, as only VEH pretreated cocaine mice move significantly more than saline control mice in the cocaine paired compartment during drug-free postconditioning test and low-dose primed reinstatement test (*F*(2,19) = 9.012, *p =* 0.0018, ****, *p* < 0.0001, ***, *p* < 0.001 compared to saline-treated mice, Bonferroni’s *post hoc* test after significant two-way repeated-measures ANOVA). ***F***, Distance traveled of every mouse during the 30 min of each conditioning session in the cocaine-paired compartment (first through fourth). VEH pretreated cocaine mice move significantly more than VEH pretreated saline mice in all sessions, show increased locomotor response to cocaine over sessions, and move significantly more than CNO pretreated cocaine mice in the later sessions (*F*_TIME_(3,57) = 10.22, *p* < 0.0001, *F*_GROUP_(2,19) = 15.21, *p =* 0.0001, and *F*_INTERACTION_(6,57) = 7.515, *p* < 0.0001, two-way repeated-measures ANOVA, Bonferroni’s *post hoc* test; **** *p* < 0.0001, *** *p* < 0.001, ** *p* < 0.01 compared to saline-treated mice and ^###^, *p* < 0.001 compared to CNO pretreated cocaine mice. Within-group comparisons to first session, **** *p* < 0.0001, *** *p* < 0.001). ***G***, Illustration of the CPP apparatus with treatment during conditioning to assess CNO-alone effects on place preference. ***H***, Time spent in the light (paired) compartment in percentage of baseline (average of pretests for each group) of saline-conditioned hM4Di mice pretreated with CNO either in both compartments or only in the gray (the latter receiving a VEH injection before saline in the dark compartment). Dashed gray bars represent the saline control group from the experiment shown in ***C*** and ***D***, included here for comparison. CNO alone had no influence on place preference, with no difference between mice pre-administered CNO in both compartments or just the one (*F*_TIME_(4,80) = 3.582, *p =* 0.0097, *F*_GROUP_(2,20) = 0.1793, *p =* 0.8372, and *F*_INTERACTION_(8,80) = 0.8799, *p =* 0.5371, two-way repeated-measures ANOVA). Data are shown as mean + SEM. Note, in these series of experiments, non-DREADD CNO controls were not included.

The acute locomotor data strongly suggest that hM4Di-mediated inhibition influences VTA neuron activity, DA release, and subsequent behavior. However, the seemingly intact cocaine sensitization supports that some effects of cocaine are intact following hM4Di-mediated inhibition, as also reflected by the increased the AMPAR/NMDAR ratio (Fig. 3*E*). We therefore speculated whether cocaine-induced reward-enhancing effects were altered on hM4Di-mediated inhibition of VTA neurons. To address this, we applied a cocaine-induced conditioned place preference (CPP) paradigm (Fig. 7*A*,*B*). Mice expressing hM4Di in VTA DA neurons were conditioned to cocaine 30 min after pretreatment with CNO or VEH. Strikingly, cocaine induced a preference for the paired compartment in both CNO-pretreated [cocaine (+ CNO)] and VEH-pretreated mice (Fig. 7*C*), compared to saline-treated mice relative to baseline levels (Fig. 7*C*; *F*(2,19) = 6.549, *p =* 0.0069, one-way ANOVA, Bonferroni’s *post hoc* test; **, *p* < 0.01, *, *p* < 0.05 relative to saline, *n*: 8 saline, 6 cocaine (+ CNO), 8 cocaine).

### Mice with hM4Di-mediated inhibition of VTA during conditioning show slower extinction of the cocaine-induced place preference

Nonetheless, during extinction, we observed a putative reward-related behavioral difference between mice that during conditioning were pretreated with CNO and VEH. That is, the CNO-pretreated hM4Di mice required more sessions before they extinguished their preference for the cocaine-paired compartment compared to baseline levels (Fig. 7*D*; *F*_TIME_(8.152) = 8.656, *p* < 0.0001, *F*_GROUP_(2,19) = 2.174, *p =* 0.1412, and *F*_INTERACTION_(16,152) = 1.833, *p =* 0.0315, two-way repeated-measures ANOVA, Bonferroni’s *post hoc* test; ****, *p* < 0.0001, ***, *p* < 0.001, **, *p* < 0.01 compared to baseline). Ten days after the last extinction session, all mice were given a priming dose of cocaine (5 mg/kg) to assess reinstatement of the previous cocaine-induced preference. Mice that were not treated with CNO during conditioning spent significantly more time in the previously paired compartment compared to pretest levels (Fig 7*D*), but there was no significant difference between the groups on reinstatement (*F*(2,19) = 1.932, *p =* 0.1723, one-way ANOVA). The distances traveled on hM4Di-mediated inhibition of the VTA neurons by CNO was also assessed during the last pretest, postconditioning, and reinstatement. Importantly, VEH pretreated (cocaine), but not the CNO-pretreated cocaine mice [cocaine (+ CNO)], moved significantly more than saline control mice in the cocaine-paired compartment during the drug-free postconditioning and low-dose primed reinstatement test, respectively (Fig. 7*E*; *F*_TIME_(2,38) = 38.60, *p* < 0.0001, *F*_GROUP_(2,19) = 9.012, *p =* 0.0018, and *F*_INTERACTION_(4,38) = 5.036, *p =* 0.0023, two-way repeated-measures ANOVA, Bonferroni’s *post hoc* test; ***, *p* < 0.001, ****, *p* < 0.0001 compared to saline-treated mice). Furthermore, during conditioning sessions, the VEH-pretreated cocaine mice moved significantly more than VEH-pretreated saline mice in all sessions, as well as significantly more than CNO-pretreated cocaine mice [cocaine (+ CNO)] in the third and fourth sessions (Fig. 7*F*; *F*_TIME_(3,57) = 10.22, *p* < 0.0001, *F*_GROUP_(2,19) = 15.21, *p =* 0.0001, and *F*_INTERACTION_(6,57) = 7.515, *p* < 0.0001, two-way repeated-measures ANOVA, Bonferroni’s *post hoc* test; ****, *p* < 0.0001, ***, *p* < 0.001, **, *p* < 0.01 compared to saline-treated mice and ^###^, *p* < 0.001 compared to CNO-pretreated cocaine mice; within-group comparisons to first session; **** *p* < 0.0001, *** *p* < 0.001). These results highlight a mechanistic dissociation in cocaine-induced responses, in which locomotor activity is highly affected by hM4Di-mediated inhibition while reward perception seems essentially unaffected.

Despite the lack of effect of CNO on cocaine-induced place preference, the extended extinction phase could be a consequence of CNO and hM4Di-mediated inhibition alone, which potentially could alter basal preference for the different compartments. Thus, to circumvent the influence by hM4Di-mediated inhibition on place preference with repeated testing, a separate cohort of hM4Di mice were pretreated with CNO before saline injections in all conditioning sessions or only light-compartment conditioning sessions (the latter receiving VEH before saline in the dark compartment; Fig. 7*G*). In these settings, CNO- and hM4Di-mediated inhibition of VTA DA neurons alone did not alter place preference. No difference in time spent (percentage of baseline) during posttest, repeated testing (i.e., extinction), and after a priming dose of cocaine (i.e., reinstatement) between these two groups or the VEH-treated saline group from the previous experiment was observed [Fig 7*H*; *F*_TIME_(4,80) = 3.582, *p =* 0.0097, *F*_GROUP_(2,20) = 0.1793, *p =* 0.8372, and *F*_INTERACTION_(8,80) = 0.8799, *p =* 0.5371, two-way repeated-measures ANOVA, *n*: 7 saline (+ CNO), 8 saline (+ CNO/VEH), and 8 saline (+ VEH)].

### Chemogenetic inhibition of VTA DA neurons decreases motivational aspects of reward but does not affect perception of reward

**Figure 8.**
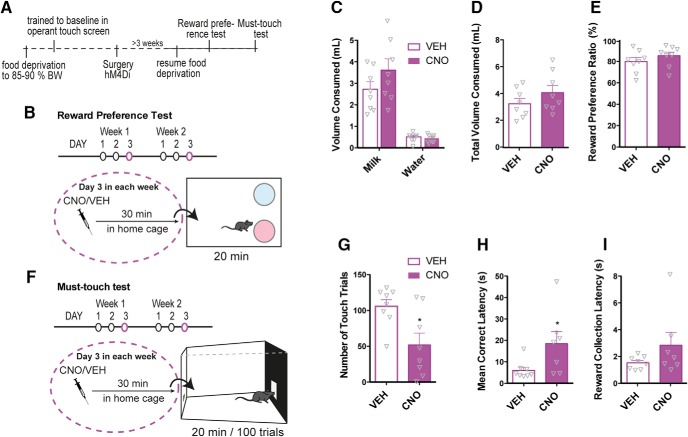
Stimulation of hM4Di in VTA DA neurons attenuates motivational aspects of reward without affecting reward perception. ***A***, Timeline of behavioral assessment of reward perception in the reward preference test (as shown in ***B***) and reward motivation in an operant must-touch test (as shown in ***F***) following CNO treatment in mice expressing hM4Di in VTA DA neurons. ***B***, Illustration of the reward preference test in which hM4Di mice for three consecutive days spent 30 min in a cage with two bowls at the rear end, one filled with water and the other with sweetened milk. On the third day, the mice were treated with VEH or CNO 30 min before the test to assess the influence of hM4Di stimulation on the consumption of water and sweetened milk. ***C***, ***D***, Volumes (ml) of water and sweetened milk consumed during the test were measured. CNO did not affect the total amount of liquid consumed (***D***; unpaired *t* test; *t*_14_ = 1.27, *p =* 0.22, *n* = 8) or preference for the reinforcer over water (***E***; unpaired *t* test; *t*_14_ = 1.08, *p =* 0.3, *n* = 8). ***F***, Illustration of touchscreen-based operational assay to assess motivation to work for the same sweetened milk reward. Touching the illuminated field on the touch screen was rewarded by a droplet of milk in the tray at the rear end. Once the reward was consumed, an area lit up again ready for another touch. The test lasted 20 min or until the mouse had completed 100 trials. Mice expressing hM4Di were tested for three consecutive days with VEH or CNO treatment on the third day. ***G–I***, CNO significantly reduced the number of touch trials (***G***; unpaired *t* test; *t*_14_ = 2.83, *p =* 0.01, *n* = 8) and mean correct latency (***H***; unpaired *t* test; *t*_14_ = 2.28, *p =* 0.04, *n* = 8) but showed no significant reduction on reward collection latency (***I***; unpaired *t* test; *t*_14_ = 1.11, *p =* 0.29, *n* = 8). Data are shown as mean + SEM. Note, in these series of experiments, non-DREADD CNO controls were not included.

To further understand the effect of hM4Di signaling in reward processing, we asked whether CNO (2 mg/kg) would alter reward-related responses to natural rewards (i.e., sugar) in a reward perception assay and a motivation-based assay (see Fig. 8*A*,*B*,*F*). We found that hM4Di-mediated inhibition of VTA DA neurons 30 min before a reward preference test (Fig. 8*B*) had no effect on total volumes consumed (Fig. 8*C*,*D*; unpaired *t* test; *t*_14 =_ 1.27, *p =* 0.22, *n* = 8) or on reward preference ratio, sweetened milk versus water (Fig. 8*E*; unpaired *t* test; *t*_14_ = 1.08 *p =* 0.30, *n* = 8). However, the same dose of CNO significantly impaired the motivation to work for that same salient reward in a touchscreen-based motivational assay, in which the mice had to touch an illuminated square to obtain sweetened milk (Fig. 8*F*,*G*; unpaired *t* test; *t*_14_ = 2.83, *p =* 0.01, *n* = 8). The latency from picking up the reward to touch the screen again was significantly increased (i.e., mean correct latency; Fig. 8*H*; unpaired *t* test; *t*_14_ = 2.28, *p =* 0.04, *n* = 8), while the latency from touching to picking up the reward (i.e. reward collection latency) was unaltered (Fig. 8*I*; unpaired *t* test; *t*_14_ = 1.11, *p =* 0.29, *n* = 8).

## Discussion

The present work substantiates that DREADDs can be a powerful tool when attempting to elucidate neuronal signaling mechanisms underlying discrete natural and drug-induced behaviors in rodents. Indeed, there is strong evidence that the synthetic DREADD agonist CNO is inert in mice, thereby allowing specific temporal activation of the expressed receptors (Armbruster et al., 2007; Roth, 2016). It should be noted, however, that recent studies have suggested conversion following *in vivo* administration in rodents of CNO to clozapine, a DA D2 receptor antagonist (MacLaren et al., 2016; Gomez et al., 2017). Gomez et al. further reported that clozapine, but not CNO, crosses the blood–brain barrier and that clozapine binds to and activates the DREADD receptors with high affinity and potency, while CNO only binds to DREADDs with low affinity (Gomez et al., 2017). Nonetheless, although this could explain, for example, the surprisingly long-lasting behavioral effects observed following CNO-mediated DREADD activation (Wang et al., 2013), the results should not affect the conclusions drawn in this present study. Here, CNO-alone controls were included in most paradigms and did not reveal any CNO effects in non-DREADD-expressing mice. Moreover, it is important to stress that the resulting concentrations of clozapine predictably are too low to affect D2 receptor function at concentrations of CNO used (Gomez et al., 2017). This is also supported by the observed intact cocaine CPP in CNO-treated mice, given that clozapine has been shown to block cocaine CPP (Kosten and Nestler, 1994). In addition, inclusion of both VEH-treated DREADD mice and WT controls, together with a thorough characterization and validation of the TH-Cre mouse (Runegaard et al., 2017), takes into account possible confounding factors and allows us to assign changes in behavior following CNO treatment in TH-Cre mice injected with hM4Di or rM3Ds to DREADD-mediated Gi or Gs-signaling in DA neurons.

Our data reveal that injection of CNO in mice expressing DREADDs in VTA DA neurons leads to prominent alterations of both natural and cocaine-induced behaviors. For natural behaviors, the results specifically demonstrated how Gi- and Gs-coupled signaling was able to bidirectionally regulate novelty-induced exploratory activity and how Gi-coupled signaling impaired motivation to work for a reward in a touchscreen-based motivational paradigm. On the other hand, hM4Di-mediated inhibition had no effect on locomotor activity following habituation or on reward preference. These results not only substantiate the major role of DA and VTA DA neurons on locomotion and reward but also suggest a putative differential role of VTA DA activity on novelty-induced and basal locomotor regulation as well as on reward perception and reward-based motivation. It is possible that these behaviors engage VTA DA neurons differently, which could interfere with the sensitivity of these DA neurons to modulatory inputs such as hM4Di, even when considering the expected massive and unspecific activation of hM4Di. In addition, when targeting VTA DA neurons that have more recently emerged as a complex heterogeneous population of neurons capable of coreleasing GABA (Tritsch et al., 2014) and glutamate (Hnasko et al., 2010), it is possible that the DREADD interventions might modulate neurotransmitters other than dopamine.

The effects on cocaine-induced behaviors were highly interesting. Stimulation of hM4Di in VTA DA neurons significantly attenuated cocaine-induced locomotor hyperactivity in mice expressing hM4Di in TH-positive neurons primarily residing in VTA. In some instances, the SN was slightly infiltrated, but we found no correlation between altered behavioral response to CNO and mice with mCherry-injected neurons in the SN. Moreover, when hM4Di expression was confined exclusively to VTA-NAc projections by use of a dual-viral approach, cocaine-induced hyperlocomotion was attenuated. This confirms that NAc acts as a primary structure for regulating cocaine-induced locomotor behavior in line with other recent reports (Boekhoudt et al., 2016) and earlier studies suggesting that NAc is primary target for cocaine-induced responses (Carboni et al., 1989; Adinoff, 2004).

It was striking that hM4Di-mediated inhibition of VTA DA neurons seemed to be specifically related to the locomotor effects of cocaine, leaving cocaine conditioning, as assessed by CPP, intact. This suggests that cocaine-induced hyperlocomotion and the reward-enhancing effects are distinct processes and might occur through different pathways, or at least that they are regulated by different mechanisms within VTA DA neurons. Such a view is supported by previous investigations segregating cocaine-induced hyperlocomotion and reward (Zweifel et al., 2008; Eisener-Dorman et al., 2011; Gifuni et al., 2012). Notably, when NMDARs were deleted specifically in DA neurons, mice had intact acute response to cocaine but did not sensitize or exhibit place preference in one-trial or classic CPP (Zweifel et al., 2008). Similarly, stimulant, hedonic, and motivational effects of cocaine can be dissociated and may, like natural rewards and novelty-induced locomotion, engage the dopamine system in very different ways, and therefore seem to be regulated differently by various signaling pathways within VTA DA neurons.

While concomitant administration of cocaine and CNO persistently attenuated cocaine-induced locomotion, we observed that CNO did not attenuate the sensitized locomotor response to cocaine in cocaine-sensitized mice. Importantly, this may reflect cocaine-induced neuronal adaptations in the VTA during withdrawal that override the ability of hM4Di-mediated inhibition to sufficiently suppress VTA DA signaling and behavior. In support of this, repeated cocaine exposure and cocaine sensitization were recently linked to increased subthreshold activity of VTA neurons (Arencibia-Albite et al., 2017), which could reflect a generally impaired function of inhibitory modulatory input to VTA. This might relate to the reduced GIRK channel-mediated currents in midbrain dopaminergic neurons observed following repeated psychostimulant exposures (Kobayashi et al., 2007; Padgett et al., 2012). Regardless of whether this effect is caused by impaired Gi-coupling due to enhanced activity of the regulator of G protein signaling (RGS) 2 (Calipari et al., 2014) or desensitization of GIRK channels, it may similarly impair the functional effect by hM4Di-mediated inhibition in VTA DA neurons. This could explain why the behavioral effect of hM4Di-mediated inhibition was absent following withdrawal after repeated cocaine injections. Desensitization of the hM4Di receptor is another possibility, but not very likely because we also observed a lack of effect in sensitized, CNO-naive animals. In addition, desensitization is unlikely given the relatively high expression levels achieved with AAV-DREADD delivery and consequently large DREADD receptor reserve (Roth, 2016). Finally, in another cohort of mice, CNO- and hM4Di-mediated inhibition had no influence on cocaine-induced locomotion when the mice had been treated with repeated cocaine in the home cage before the test. Therefore, the lack of effect of hM4Di-mediated inhibition following repeated cocaine conceivably reflects endogenous adaptations in VTA DA regulation, adaptations that seem to be independent on associated cues and context. Accordingly, cocaine-induced potentiation (Ungless et al., 2001) was not prevented by hM4Di-mediated inhibition before cocaine, as we found that the AMPAR/NMDAR ratio in VTA neurons increased independently of CNO. This indicates that some effects of cocaine are intact and that adaptations occur despite hM4Di-mediated inhibition and do not involve a general alteration in DA homeostasis as a consequence of repeated CNO- and hM4Di-mediated inhibition in VTA DA neurons. In summary, the experiments show that repeated cocaine administration leads to a sensitized locomotor response to cocaine after a withdrawal period, independent of whether the locomotor-stimulant effect was attenuated during sensitization.

Chemogenetic stimulation of Gi-signaling in hM4Di-expressing neurons is obviously artificial. It should also be considered that precise subcellular specified signaling to specific somatodendritic or axonal domains is not allowed, rather only proximately if using DREADD-fusion constructs (Dong et al., 2010). For this reason, hM4Di stimulation is usually associated with a general neuronal inhibition, but given the metabotropic nature of DREADDs, the effect of CNO in hM4Di mice may be highly dependent on the overall excitation level of the neurons, besides the simultaneous modulatory and regulatory inputs received by the neurons. This is interesting for the interpretation of the chemogenetic intervention and should be considered given the complexity of the results—specifically, the impaired effect of CNO after repeated cocaine. Our data indicate a cocaine-mediated impairment of the effect of CNO in hM4Di mice, and that this effect is not context related. Although representing a potential caveat on the use of chemogenetics to inhibit neuronal activity, it also provides useful and biological relevant information on the impact cocaine has on VTA DA signaling and regulation. In short, it seems that repeated cocaine can disrupt the sensitivity to metabotropic inputs of VTA DA neurons.

Nonetheless, the major impact by hM4Di-mediated inhibition on the acute response to cocaine suggests significant metabotropic capacity of VTA DA neurons to prevent the acute effect of cocaine, which is believed to be due to DAT blockade at the terminal (as well as cell bodies), leading to significant increase in dopamine. Interestingly, both somatodendritic GABA_B_ and dopamine D2 autoreceptors, among others (e.g., 5-hydroxytryptamine, muscarinic, and endocannabinoid receptors), on VTA neurons signal via Gi-coupled receptors and compromise a significant endogenous modulatory input to VTA DA neurons (Ford, 2014; Johnson and Lovinger, 2016). Indeed, Gi-coupled GABA_B_ receptors expressed on VTA dopaminergic neurons were shown to mediate a GABAergic input from NAc medium spiny neurons that regulate cocaine-induced hyperlocomotion (Edwards et al., 2017). Acute conditional genetic deletion of GABA_B_ receptors in VTA neurons increased the acute locomotor response to cocaine (Edwards et al., 2017), and similar findings were obtained on conditional genetic deletion of D_2_ autoreceptors (Bello et al., 2011). These selective receptor deletion studies support our findings of a substantial inhibitory influence by Gi-signaling in mesolimbic DA neurons on acute cocaine-induced behaviors.

As revealed by a series of DA lesion studies, the “liking” part of reward processing, at least for natural rewards, seems independent of DA (Berridge et al., 1989). This has led to the hypothesis that DA adds incentive value, a motivational drive to obtain a reward (Berridge and Robinson, 2016; Koob and Volkow, 2016). Our data directly support this hypothesis, showing that during hM4Di-mediated inhibition in VTA DA neurons, the motivation to work for a hedonic reward (sweetened milk) in a learned must-touch assay is suppressed, while the hedonic value of the reward itself remains intact. It is tempting to elaborate this hypothesis further in relation to the cocaine-induced responses observed in our CPP paradigm. Thus, the absent effect of hM4Di-mediated inhibition on cocaine conditioning could reflect intact reward perception of cocaine, and the extended extinction phase could reflect altered encoding of the incentive value of cocaine and associated cues. It is therefore tempting to propose a mechanism by which hM4Di-mediated inhibition, in VTA DA neurons, reduces VTA activity and DA release in NAc, impairing cocaine-induced increase in DA levels. Consequently, this directly affects locomotor activity but also impairs the encoding of incentive value to cocaine-paired cues. The latter may explain the prolonged extinction, given the role of DA signaling as a vital learning system based on reward prediction errors that guide responses according to environmental stimuli (Schultz et al., 1993; Schultz, 2006). On lack of expected reward, negative DA signals, so-called negative prediction errors, should facilitate adjustment of behavior. Thus, if hM4Di-mediated inhibition impairs the encoding of incentive value during cocaine conditioning, it is plausible that these mice respond with smaller negative prediction error signals on lack of reward during extinction and therefore have lower motivation to change behavior (i.e., they return to the preferred dark CPP compartment).

In conclusion, by employing a chemogenetic approach, we substantiate DA as a prime regulator of reward motivation, as well as reveal a complex and delicate key role of metabotropic Gi-coupled signaling in regulating VTA DA output with differential effects during acute, repeated, and sensitized responses to an addictive drug such as cocaine. In this way, the data may provide an important framework for exploring novel strategies and principles for treatment of addiction via modulation of GPCR signaling cascades within the dopaminergic system.

**Table 1.**
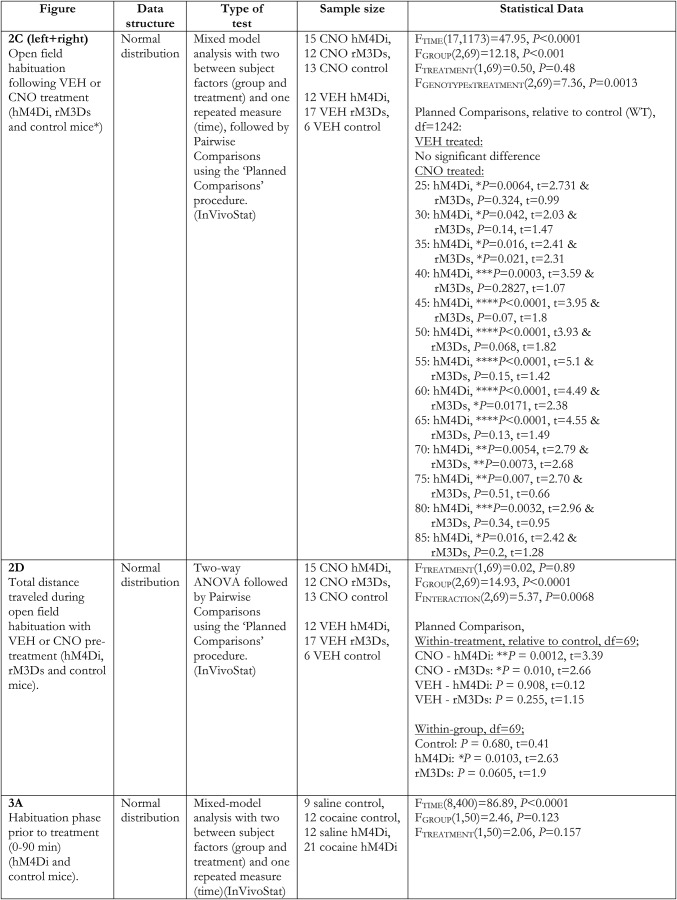

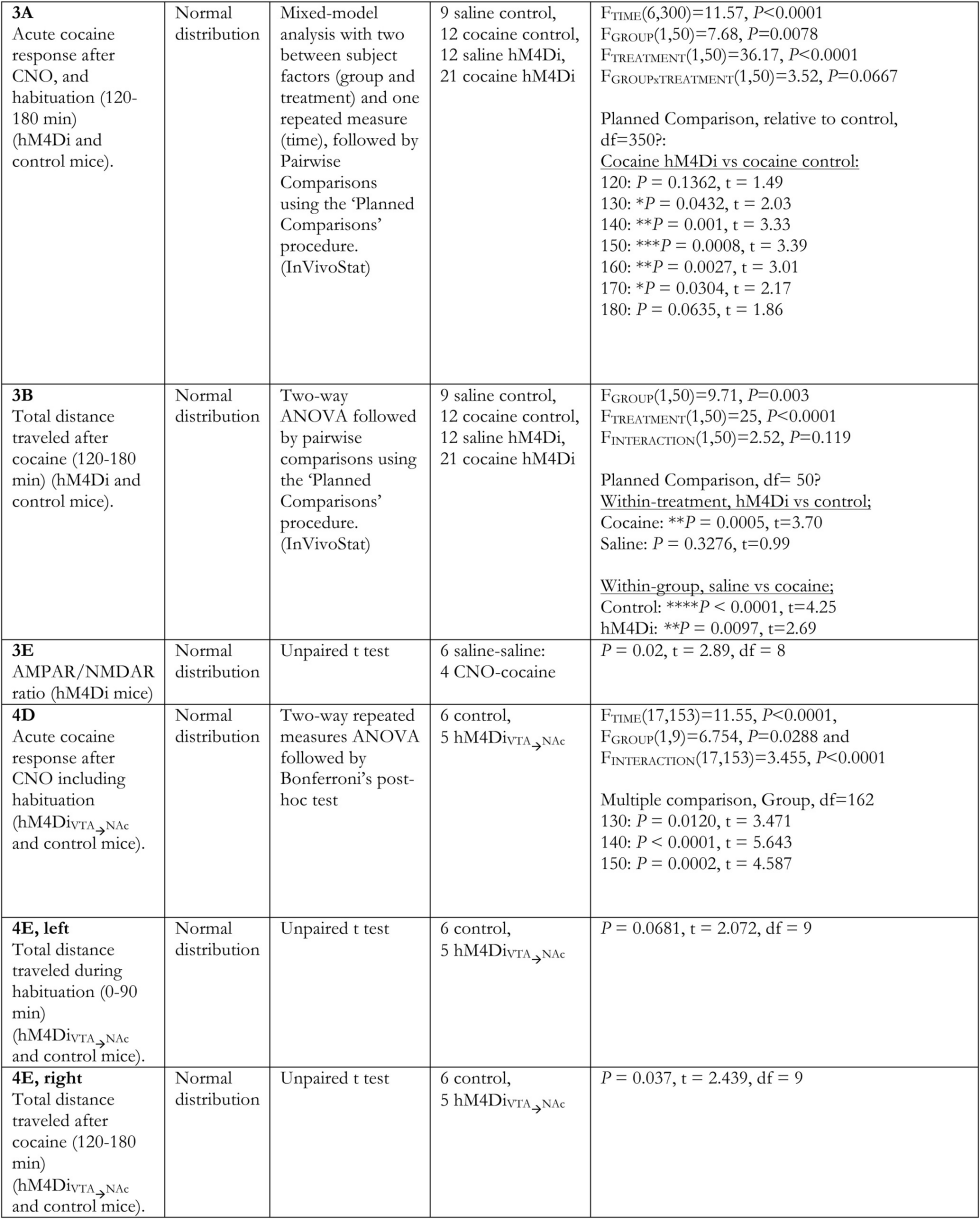

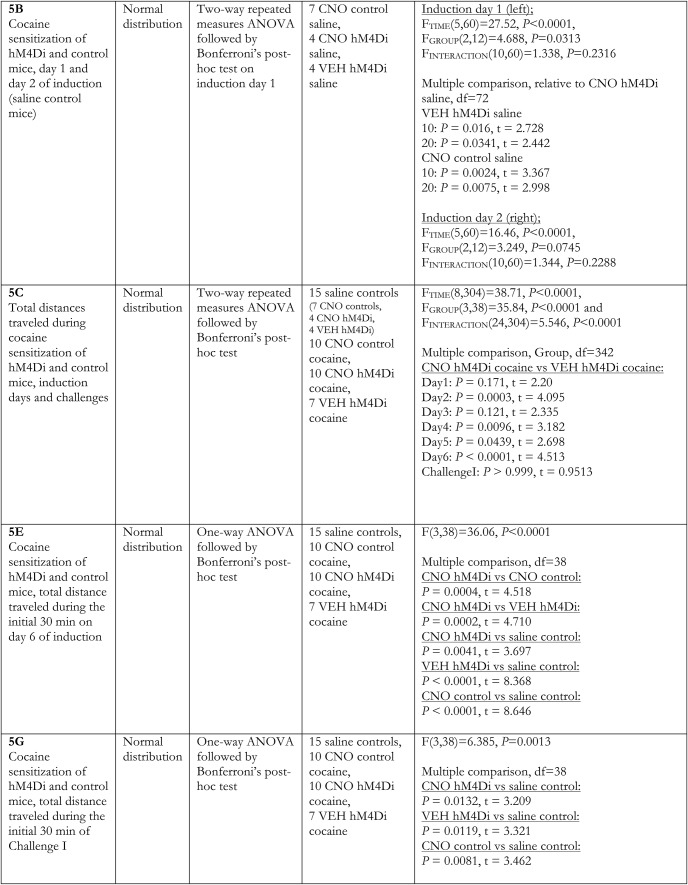

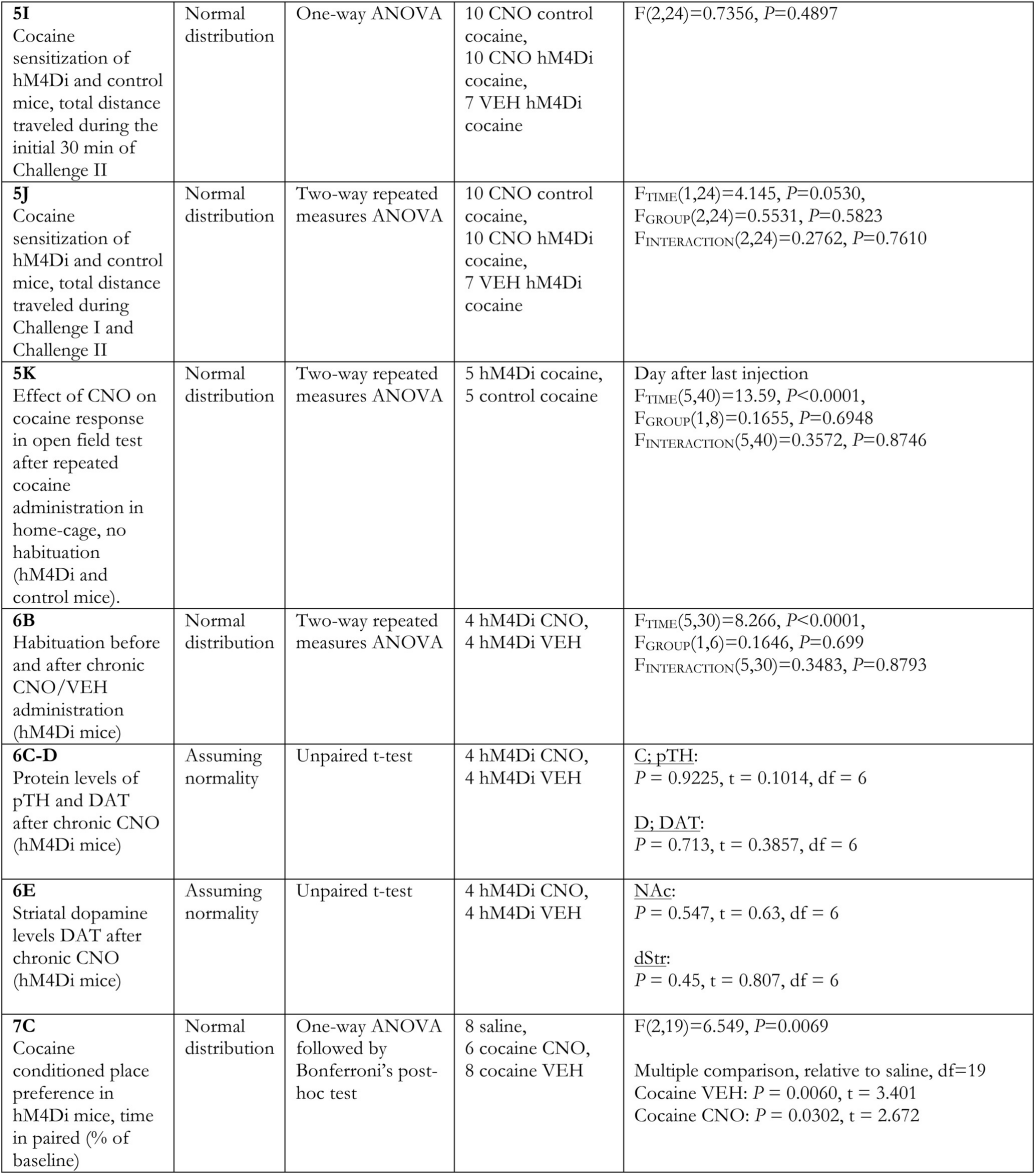

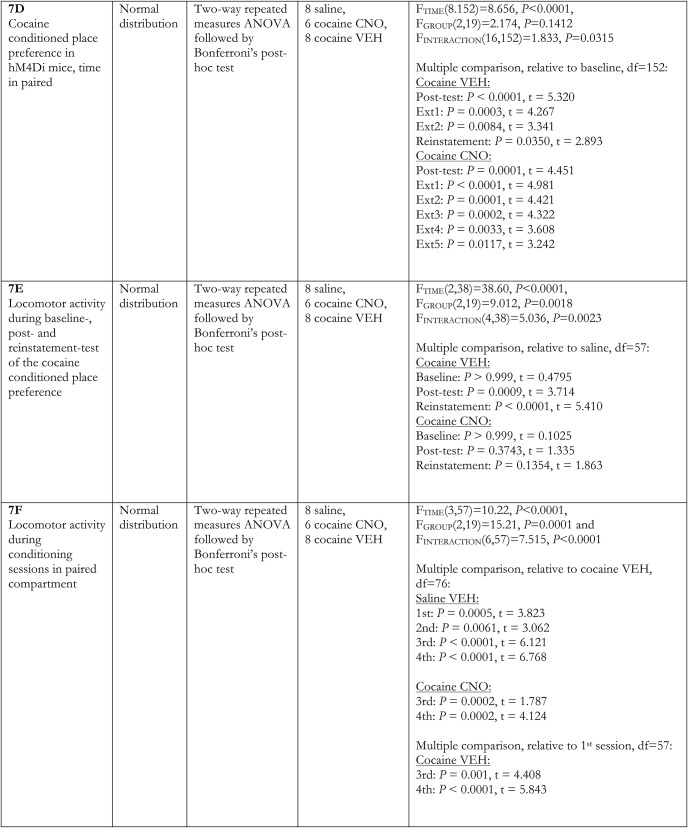

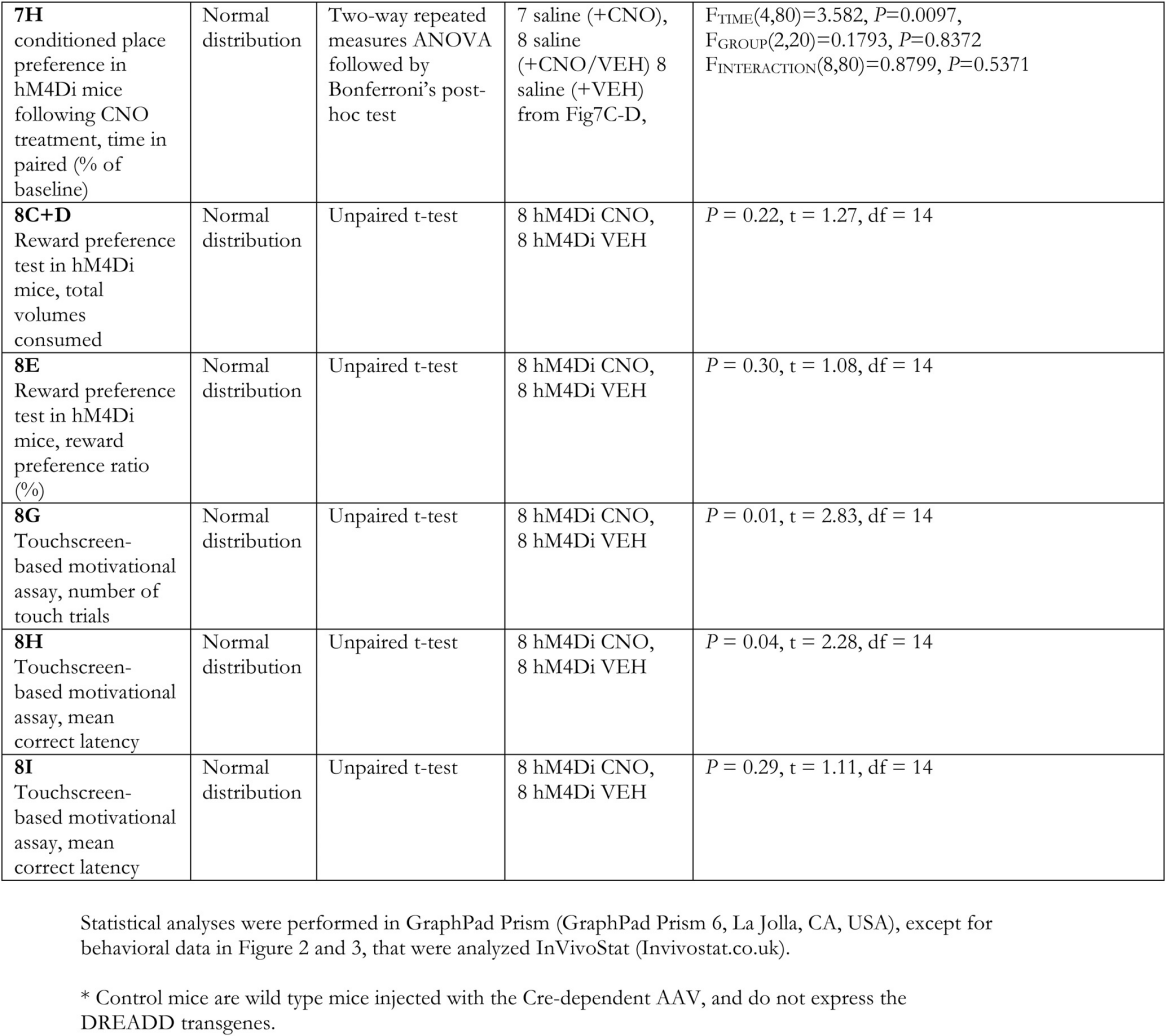
Statistical analysis

## Synthesis

Reviewing Editor: Karen Szumlinski, University of California at Santa Barbara Decisions are customarily a result of the Reviewing Editor and the peer reviewers coming together and discussing their recommendations until a consensus is reached. When revisions are invited, a fact-based synthesis statement explaining their decision and outlining what is needed to prepare a revision will be listed below. The following reviewer(s) agreed to reveal their identity: Ryan LaLumiere.

Upon consultation with the original two experts reviewers of article, both reviewers found the revised version much improved. However, we concur that further revision is required before your report is suitable for publication in eNeuro. More specifically, the statistical analyses continue to be a major problem because the authors tend to collapse 2 independent factors (drug: (either CNO/VEH or SAL/COC and genotype: hm4di/WT/rm3ds and refer to this collapsed factor as ‘group’). This is incorrect - new analyses must be performed. Also, the authors do not always compare the necessary groups. For example, in Fig. 2 it is essential that the authors compare CNO vs. VEH in the same genotype and they never do that. This is critical as it could be constitutive activity that is leading to the variation in activity rather than a CNO-specific effect. Given the number of statistics presented in the manuscript, we strongly recommend that the authors recruit a statistician to help them run the appropriate analyses.

## Author Response

Response to Reviewers: We are pleased to read that you and the two reviewers found potential interest in our work and acknowledge the scientific significance of our manuscript. We appreciate the insightful comments from the reviewers. In the revised manuscript, we now include an extra patchclamp electrophysiology experiment and two additional behavioral experiments to provide the requested controls. We find that the substantial revision of the manuscript according to the reviewer's suggestions has not only strengthened the manuscript overall but also specifically strengthened the validity of selective CNO-mediated effects and led to supportive evidence for a significant attenuation of chemogenetic Gi-signaling in VTA neurons following cocaine exposure.

Please read our point-by-point response below, with references to specific manuscript changes, which in the revised manuscript are marked with red font.

Reviewer 1: “1. The electrophysiology data with hM3Dq that was mentioned in results should be show in Figure 1. In addition, given the recent publications regarding non-DREADD effects of CNO, electrophysiology data showing no effect of CNO in controls should also be included.”

We acknowledge the missing control in Figure 1G and have performed additional recordings on slices from mice transduced with a control vector expressing Cre-dependent mCherry only (Figure 1, panel G). We now show that local CNO puff in coronal midbrain sections from mice transduced with hM4Di decreases neuronal spike activity in mCherry/TH-positive neurons, while there is no effect of CNO in mice transduced with the control vector. We consider this as a better control than hM3Dq to reveal CNO specificity. Since other hM3Dq experiments are not included elsewhere in the manuscript, we believe it would be more distracting than helpful to show the hM3Dq trace why we have decided not to mention hM3Dq recordings in the revised manuscript. The data of the mCherry control, including methods, have been incorporated in the Methods section (p. 8-9), Results section (p. 18), and Figure 1 legend (p. 40-41).

“2. It would be helpful to include individual data points in all of the bar graphs to get an idea of variance.”

2

We acknowledge this comment and have now included data points in bar graphs (Fig. 2, Fig. 3, Fig. 5, Fig. 7 and Fig. 8). The only exception is the graphs showing CPP data with multiple columns (Fig. 7) where it became more distracting than helpful.

“3. The statistical analyses that were performed aren't always clear, and they should be included in the results section (not just the figure legends). For RM ANOVA, what the factors for analysis were and any main effects should be reported. For the data in Figure 2, were the CNO and VEH pretreatment groups analyzed together - if not, this should be addressed.”

As requested by the reviewer, we now include the statistical analysis in the Results section. Missing factors for analysis and main effects are also reported (p. 19, p. 20, p. 22, p. 23, p. 24, p. 25, p. 26, p. 27 and p. 28).

For the data in Figure 2, CNO and VEH treated groups were analyzed together in an initial RM ANOVA revealing that in rM3Ds-expressing mice, CNO pre treatment increased locomotion during habituation as compared to WT control mice (FTIME(17,1173)=86.14, P&lt;0.0001, FGROUP(5,69)=8.017, P&lt;0.0001 and FINTERACTION(85,1173)=1.927, P&lt;0.0001, two-way repeated measures ANOVA, Bonferroni's post-hoc test; **P&lt;0.01 rM3Ds-CNO vs control-CNO and P=0.062 hM4Di-CNO vs control-CNO, N: 15 hM4Di CNO, 12 rM3Ds CNO, 13 control CNO, 12 hM4Di VEH, 17 rM3Ds VEH, 6 control VEH). However, in this multiple group analysis we would like to point out that the hM4Di-CNO vs control-CNO comparison did not reach our predefined criteria for significance (p &lt; 0.05), but was approximate the same (P = 0.0616). If excluding the last phase of the habituation (75-90 min interval) where locomotion in all groups was much reduced (except the rM3Ds-CNO), the hM4Di-CNO group display significantly lower locomotion than the control-CNO group (Locomotion0-75min; FTIME(14,966)=77.02, P&lt;0.0001, FGROUP(5,69)=8.763, P&lt;0.0001 and FINTERACTION(70,966)=1.829, P&lt;0.0001, two-way repeated measures ANOVA, Bonferroni's post-hoc test; **P&lt;0.01 rM3DsCNO vs control-CNO and *P&lt;0.05 hM4Di-CNO vs control-CNO, N: 15 hM4Di CNO, 12 rM3Ds CNO, 13 control CNO, 12 hM4Di VEH, 17 rM3Ds VEH, 6 control VEH). Thus, we reasoned that further analysis was required for a fair presentation of the data. Instead of showing all 6 groups in one figure and perform the analysis on the initial habituation phase (e.g. 0-75min), we decided to present the CNO- and VEH-pretreated mice in separate figures and assess locomotion and time by time differences between control mice and hM4Di/rM3Ds mice with

3

similar pre-treatment. We clearly state this in the manuscript, and report its full analysis, including factors on page 19-20.

“4. There are some inconsistencies with baseline/novelty locomotor activity across experiments that needs to be addressed. In the acute cocaine experiment, it appears that the saline hM4Di group has a significantly different locomotor response during habituation compared to the other groups. This should be described and discussed particularly because the magnitude of the difference between this group and the other groups (e.g. cocaine control) looks the same as what was reported with CNO in Figure 2. Also, the authors should include a justification for the large difference in sample size across groups for this experiment - the variance in the cocaine control is very large relative to the cocaine hM4Di group and sample size could be a factor in that. This same point is relevant for the Figure 4 data, as it looks like locomotion is different in the hM4Di animals prior to CNO (constitutive activity changes?) - which makes the cocaine effects challenging to interpret, especially without saline/saline controls.”

We agree that the graphic illustrations might give the impression that some baselines appear inconsistent. However, statistical analysis of the habituation phase (total distance traveled 0-90 min) in the acute cocaine experiment (Figure 3) does not reveal any significant difference between the saline hM4Di and any of the other groups (F(3,50)=1.52, P=0.221, one-way ANOVA). Further analysis and assessment of individual time points of the habituation phase (090 min) by RM 2-way ANOVA revealed no overall difference between groups (FGROUP(3,50)=1.520, P=0.221, two-way repeated measure ANOVA). The same is true for data in Figure 4. Here the difference between groups is not significant until after cocaine administration as reported in the figure. Also when analyzing the habituation phase (total distance traveled 0-90 min), the WT control are trending towards, but does not display significantly higher locomotor activity than hM4Di(VTA→NAc) mice (t(9)=2.072, p=0.0681, unpaired t-test). These considerations are now described and discussed on page 20 and 23 of the revised manuscript.

The reason for sample size differences across groups was because we included 2-3 extra DREADD mice than control mice in each cohort. This was done empirically in case we had to exclude some DREADD mice when determining viral expression patterns after end experiment. This is now mentioned in the Method section on page 7.

4

The reviewer correctly notices a greater variance in the cocaine control group. One factor is that some mice are low/non-responders. In the cocaine control group, such low/non-responders can affect the variance but cannot fairly be removed when comparing with the hM4Di cocaine mice where the cocaine-induced locomotor activity is diminished. Obviously, low/non-responders are hard or impossible to identify and also have less influence on variance in this group. This issue should be clearer to the reader now when the data points are added in the graphs.

“5. In addition, it sounds like induction day 1 for the sensitization experiment is actually day 2 as the same animals were used for the acute cocaine experiment. If so, this should be clarified in the experimental design shown in figure 5. Nonetheless, it doesn't look like any of the groups developed sensitization during the induction phase, this should be addressed. In addition, without the habituation phase, CNO would be expected to effect locomotion as in Figure 2 - this should be addressed by separating out the 3 vehicle control groups. If the data in induction day 1 does not replicate the novelty locomotor experiment, this should be discussed.”

It is correct that the mice in the sensitization experiment were used for the acute cocaine experiment. This was already described in the method section and has now been mentioned again in the Results section (p. 22). As requested by the reviewer, it has also been included in the experimental design shown in Figure 5A.

As also correctly noticed by the reviewer, the mice do not show a clear increase in locomotor activity during the induction phase of sensitization, which may be explained by the experimental design and the previous exposure to cocaine. We now address this in the Results section (p. 22).

Given our observations in previous experiments in the open field paradigm, we agree with the reviewer that CNO treatment in hM4Di mice prior to habituation would be expected to affect locomotion. However, no difference between saline groups was found in the overall statistic analysis on any days of the behavioral sensitization (FGROUP(2,12)=0.475, P=0.633, FTIME(8,96)=28.34, P&lt;0.0001, FINTERACTION(16,96)=0.240, P=0.999). This was the rationale for pooling these saline controls. Nevertheless, further analysis and comparison of saline group mice on day 1 of induction did reveal a slight but still significant reduced locomotor activity in hM4Di mice pre-treated with CNO during the initial phase of the habituation (FGROUP(2,12)=4.688, P=0.031, two-way repeated measure ANOVA, Bonferronis post-hoc test; #P&lt;0.05 compared to

5

VEH hM4Di , ** P&lt;0.01 compared to CNO control, N: 7 CNO control, 4 VEH hM4Di, 4 CNO hM4Di (see new panel C in Figure 5). While the total distance traveled during the initial 30 min was lower in CNO treated hM4Di mice on induction day 1 (F(2,12)=4.883, P=0.0281, oneway ANOVA, Bonferroni's post-hoc test; *P&lt;0.05 as compared to CNO control, P= 0.0613 as compared to VEH hM4Di,), the difference disappeared on induction day 2 (F(2,12)=3.286, P=0.073, one-way ANOVA). At this point, the activity boxes do not seem to induce much novelty-induced locomotion, and the control mice did not move more than hM4Di mice pretreated with CNO. This supports our hypothesis that VTA Gi-signaling affects novelty-driven locomotion but do not impair low basal locomotion. At the same time, the chance of detecting a significant difference is reduced as locomotor activity gradually declines.

In the manuscript, the total distance traveled of individual saline groups on induction day 1 is now shown in a revised Figure 5B. In a new panel C, the habituation on induction day 1 and 2 of individual saline groups are added. As no difference was observed between saline groups from induction day 2 and on, these groups were pooled and shown in Figure 5B from induction day 2 as one group - saline controls. The text has been revised on page 23. “6. For the acute cocaine experiment, it looks like the hM4Di animals have an attenuated but not completely blocked cocaine response. Thus, it would be interesting to determine if the AMPA/NMDA ratios are also attenuated (compare to cocaine control) rather than if greater than saline.”

We agree with the reviewer that it would be interesting to examine if the AMPA/NMDA ratio is attenuated compared to cocaine control. However, the intention of this experiment was to assess whether or not hM4Di-mediated inhibition blocked potentiation. Even if the hM4Di-mediated inhibition attenuates cocaine-mediated potentiation, it seems not to affect subsequent cocaineinduced plasticity, given the complete behavioral sensitization to cocaine observed in mice pretreated with CNO. In addition, the observed increase in AMPA/NMDA ratio is comparable to what has previously been reported following cocaine in WT mice (Ungless et al., 2001; Saal et al., 2003). Therefore, we reason that including such experiment would not affect the overall conclusions of the paper.

“7. More details regarding the criteria for viral inclusion criteria should be included. Are the images shown in Figure 1 representative or was there variance across animals? Was

6

there a minimum number of cells that needed to be observed to be included in the experiment? Also, it should be clarified how viral assessment was made in the chronic CNO animals that underwent HPLC.”

As requested by the reviewer, we have now specified how viral assessment was used as criteria for inclusion/exclusion, which also clarifies how viral assessment was made in the chronic CNO animals (p. 8). In total, 4 out of 145 mice were excluded due to lack of expression. In general, little variance across TH-Cre positive animals was observed and the images shown in Figure 1 are representative of mCherry spread and intensity in VTA.

For the chronic CNO animals that underwent HPLC analysis, viral assessment of the midbrain was carried out as for the other mice. The brains were dissected and while the striatal slices and tissue-punches were obtained for HPLC, the posterior part of the brains containing the midbrain region was immersion-fixated in 4% PFA and processed as described in the method section.

“8. The CPP data - the extinction lacks a critical control of saline + CNO to determine if CNO alone effects chamber preference and extinction. In addition, the variance is high in this experiment, it would be useful to track individual animals to determine if all animals showed impaired extinction or just a subset. The stats for the extinction phase should be clarified, which 2 way ANOVAs were performed and what were the factors?

There do not appear to be differences between cocaine/veh and cocaine/CNO groups during extinction and reinstatement of these groups does not appear different, nor does it appear different from the last day of extinction. Comparing all of the data relative to baseline is somewhat misleading.”

As requested by the reviewer, we have performed a new experiment to assess the influence of CNO alone on place preference,. Specifically, we repeated the CPP protocol with hM4Di mice that were pre-administered CNO prior to saline injections in either both chambers or only in the paired (the latter was pre-administered VEH prior to saline in the un-preferred). These results are now included in Figure 7, new panel G and H and discussed in the manuscript (p. 27). This experiment demonstrates that CNO in saline controls does not influence place preference. Furthermore, CNO (and hM4Di-mediated inhibition) in the least preferred chamber does not induce aversion or alter preference in any other way with repeated testing like in an extinction

7

phase. Likewise, no effect was observed between these mice following cocaine administration like in a reinstatement test.

As also suggested by the reviewer, we have now further tracked individual mice to rule out the cause of variation. Since we found no clear indication that only subset of mice showed impaired extinction, we chose to show the data as already presented in Figure 7D.

The statistics for the extinction has been clarified on page 26. Here we report: A two-way repeated measure ANOVA was performed (FGROUP(2,19)=2.174, P=0.1412, FTIME(8,656)=1.833, P&lt;0.0001, FINTERACTION(16,152)=1.833, P=0.0315, ****P&lt;0.0001, ***P&lt;0.001, **P&lt;0.01 *P&lt;0.05 compared to baseline, Bonferroni post-hoc test after significant two-way repeated measures ANOVA).

“9. Given recent reports that VTA dopamine neurons also co-release GABA, the authors should temper their statements throughout the manuscript that their manipulations are modulating VTA dopamine release. The possibility of modulating GABA release should also be included in the discussion”.

This is a valid point. We now mention in the Discussion section the complex and emerging evidence of a heterogeneous population of VTA dopamine neurons with co-release of GABA and glutamate, and state the possibility that the DREADD intervention might modulate other neurotransmitters than dopamine (p. 30 and 31).

“10. Many discussion points are included in the results section, these should be moved to the discussion.”

We have accommodated this suggestion as much as possible throughout the Results section.

Reviewer 2: “1. The authors do a good job of using CNO-alone controls throughout the first set of experiments, acknowledging evidence that CNO alone can alter behavior. However, I think it is important for the manuscript to explicitly note when the CNO-alone control groups were *not* used (e.g., Figure 8) and acknowledge this possible explanation for these findings (even if unlikely).”

8

We thank the reviewer for raising this point. It is true that while the first set of experiments include CNO-alone control groups, these were left out in the later experiments, based on the lack of effect in the initial experiments. Like the reviewer recognize, it is unlikely that CNOalone effects may explain the results in Figure 7 and 8.

Based on our observations on locomotion and the recent study published by MacLaren (MacLaren et al., 2016), that included a thorough assessment of non-DREADD effects with various doses of CNO, it is reasonable to conclude that our used dose of 2 mg/kg is unlike to cause the observed effects by acting on non-DREADD targets. These concerns were already mentioned in the discussion of the first version of our manuscript (p. 30) and while CNO-alone controls are included in most experiments, we now explicitly mention when they were not included in individual experiments (Fig. 6, Fig. 7 and Fig 8).

“2. While the authors speculatively explain the complexity of the results through discussion of locomotor vs. motivational vs. pleasure or hedonic effects, there are other possibilities that should be considered. The authors themselves note that cocaine may alter GPCR-mediated signaling in the neurons. As a consequence, chemogenetic manipulations would no longer function in the same way as in a naïve animal. This is a technical issue and even a technical problem - that is, chemogenetics is frequently used and described as a way to “activate” and “inhibit” cells. The authors should consider noting that changes in intracellular GPCR functioning may make interpreting chemogenetic experiments difficult. (Indeed, if the authors are correct, this creates a significant problem in chemogenetic approaches in general.)”

“Additionally, the authors do not consider that the complexity of the results may interact with how the dopamine neurons are being activated or inhibited. The DREADDs are expressed in the cell bodies and the terminals, meaning systemic injections are not targeting one specific part of the neuron. In contrast, much of cocaine's effects are believed to be due to DAT blockade at the terminal, leading to significant increase in dopamine. However, cocaine can also have effects on the VTA cell bodies (directly and indirectly - it gets complicated). Natural rewards and novelty-induced locomotion likely engage the dopamine system in completely different ways from cocaine. The authors should discuss acknowledge these points”.

9

These insightful comments of the reviewer are acknowledged and appreciated. In the revised manuscript, we now further elaborate and discuss the subcellular effects of hM4Di-mediated inhibition and its impact on various behaviors (motivation, liking, locomotion) on p. 32-33. In addition, to further determine the impact of cocaine on subsequent hM4Di-mediated inhibition of VTA DA neurons, we have carried out an additional behavioral experiment. Here, the effect of CNO and chemogenetic manipulation was assessed in a novel open field following repeated cocaine injections in home-cage (1 daily injection for 6 consecutive days with last injection administered 1 week before the open field test). In contrast to the effect of CNO in cocainenaïve mice that resulted in significant differences between hM4Di and WT mice in both habituation and the acute response to cocaine, we observed no difference between these groups of mice when tested after repeated cocaine (Figure 5K, Methods p. 11, Results p. 24). This supports our hypothesis of a cocaine-mediated impairment of the effect of CNO in hM4Di mice. It further suggests that the “lack of effect” is not context related. Despite representing a potential caveat on the use of chemogenetics to inhibit neuronal activity, it might indeed also provide useful and biological relevant information on the impact cocaine has on VTA DA signaling and regulation. Thus, the data suggest that repeated cocaine stimulations disrupt the sensitivity to metabotropic inputs of VTA DA neurons. This is addressed in the Discussion section on p. 33.

“3) While most of the manuscript is written reasonably well, the Methods section needs some improvement. Various issues with grammar repeatedly occurred”.

We have been through the Method section, which should now be of similar standard as the rest of the manuscript.

“4) The use of the term “hM4Di” stimulation can be confusing as stimulating these DREADDs inhibits the neurons. Perhaps the authors could use “hM4Di-mediated inhibition”?”

We have changed the term “hM4Di stimulation” into “hM4Di-mediated inhibition” as suggested by the reviewer and the inhibiting effect following hM4Di-activation should now be more clear to the reader.

10

“5) Figure 1 needs a time scale for panel G.”

The time scale is now shown in Figure 1 G together with a representative trace from the mCherry control mice. Thanks for pointing out.

“6) Page 20, last sentence: It should read “either . . . or” (and not “neither . . . nor”)”

This has been corrected, thanks.

General note: Two researchers, performing some of the additional experiments have been included as co-authors.

References: MacLaren DA, Browne RW, Shaw JK, Krishnan Radhakrishnan S, Khare P, Espana RA, Clark SD (2016) Clozapine N-Oxide Administration Produces Behavioral Effects in LongEvans Rats: Implications for Designing DREADD Experiments. eNeuro 3. Saal D, Dong Y, Bonci A, Malenka RC (2003) Drugs of abuse and stress trigger a common synaptic adaptation in dopamine neurons. Neuron 37:577-582. Ungless MA, Whistler JL, Malenka RC, Bonci A (2001) Single cocaine exposure in vivo induces long-term potentiation in dopamine neurons. Nature 411:583-587.
